# Rocket‐Inspired Sequentially Targeted Nanotherapeutics for Mitochondrial Regulation and Inflammatory Reprogramming in Ischemic Stroke

**DOI:** 10.1002/advs.76859

**Published:** 2026-07-27

**Authors:** He Bai, Zihao Yong, Yang Li, Qingmin Chen, Yong Liu, Xiaomeng Guo, Fukai Li, Shicheng Tang, Zhuo Yang, Yufei Zhang, Ruwei Jie, Xiaohong Lv, Huacheng Wang, Chunming Huang, Liwei Xie, Jianqi Xiao, Qingchun Mu, Longguang Tang

**Affiliations:** ^1^ Affiliated Gaozhou People's Hospital Guangdong Medical University Maoming Guangdong China; ^2^ College of Life Sciences Mudanjiang Medical University Mudanjiang Heilongjiang People's Republic of China; ^3^ Department of Pharmacy Center for Regenerative and Aging Medicine and International School of Medicine International Institutes of Medicine The Fourth Affiliated Hospital of School of Medicine Zhejiang University Yiwu China; ^4^ Shanxi Province Cancer Hospital/Shanxi Hospital Affiliated to Cancer Hospital Chinese Academy of Medical Sciences/Cancer Hospital Affiliated to Shanxi Medical University Taiyuan Shanxi China; ^5^ Department of Physiology School of Basic Medical Sciences Guilin Medical University Guilin Guangxi China; ^6^ Faculty of Synthetic Biology Shenzhen University of Advanced Technology Shenzhen China; ^7^ Department of Neurosurgery Affiliated Hospital of Guangdong Medical University Zhanjiang Guangdong China

**Keywords:** cerebral ischemia‐reperfusion injury, drug delivery, mitochondrial targeting, nanomaterials, neutrophil hitchhiking

## Abstract

Mitochondrial dysfunction causes inflammatory cascades in cerebral ischemia‐reperfusion injury (CIRI). However, precise pharmacological interventions are hindered by the restrictive blood‐brain barrier (BBB). Here, we report a neutrophil‐hitchhiking, biomimetic nanoplatform (NeuM@Mdivi‐1) with a “rocket‐inspired” sequential targeting strategy for restoring mitochondrial homeostasis. By co‐opting the innate chemotaxis of circulating neutrophils, NeuM@Mdivi‐1 effectively bypassed the BBB to infiltrate the ischemic penumbra. Within this pathological microenvironment, upregulated matrix metalloproteinase‐9 (MMP9) triggers surface transformation, exposing mitochondrial‐targeting peptides and enabling the precise intracellular release of the fission inhibitor Mdivi‐1. This spatiotemporal delivery approach effectively suppresses aberrant dynamin‐related protein 1 (Drp1)‐mediated fission, thereby silencing the Drp1/mtDNA/cGAS‐STING signaling axis and mitigating neuronal ferroptosis. NeuM@Mdivi‐1 preserves neuronal viability and reengineers the immune microenvironment by decoupling mitochondrial fragmentation from the inflammatory response, thereby establishing a sophisticated therapeutic paradigm for CIRI management.

## Graphical abstract

A novel neutrophil‐hitchhiking, rocket‐inspired nanoplatform is developed to cross the blood‐brain barrier for sequential, spatiotemporal drug delivery. By responsive surface transformation in the ischemic penumbra, it precisely targets mitochondria to suppress Drp1‐mediated fission. This elegant approach silences the Drp1/mtDNA/cGAS‐STING axis, mitigating neuronal ferroptosis and effectively reprogramming the inflammatory microenvironment during stroke management.

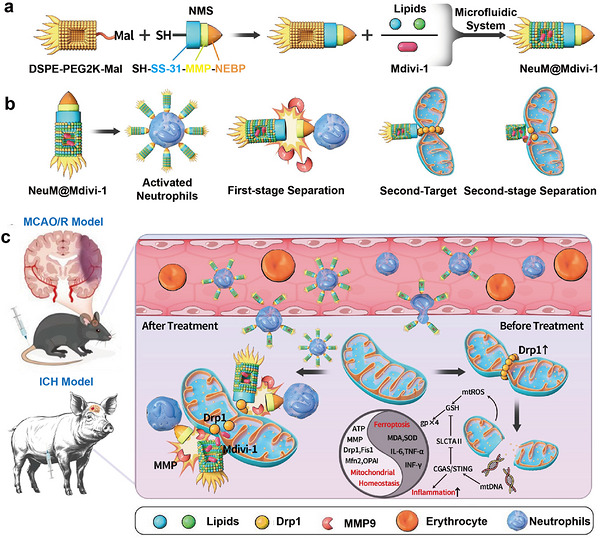

## 1 Introduction

Cerebral infarction is the second leading cause of death worldwide, and reperfusion therapy is the primary treatment strategy [1]. However, reperfusion can exacerbate neuronal injury through several cellular changes. Disrupted mitochondrial homeostasis is a key factor, driving oxidative stress, inflammation, and ferroptosis [2, 3]. Changes in mitochondrial dynamics are the initial response to mitochondrial stress, enabling cells to resist external stimuli through a continuous cycle of fission and fusion. The cerebral ischemia‐reperfusion pathology includes excessive mitochondrial fission that reduces the number of healthy mitochondria to a critical level, thus impairing their ability to protect themselves from damage [4]. This results in the irreversible accumulation of mitochondrial DNA (mtDNA), which is detected and bound by cGAS, a member of the nucleotidyl transferase family. This activates STING and the subsequent production of inflammatory cytokines and chemokines [5]. Consequently, this cascade promotes apoptosis and worsens the neuroinflammatory response associated with cerebral ischemia‐reperfusion injury (CIRI) [6]. Mitochondria are energy‐supplying organelles that are crucial for cellular metabolism and function [7]. Abnormal mitochondrial dynamics and dysfunction increase the susceptibility to ferroptosis. Mitochondrial reactive oxygen species (mtROS) release during fission substantially contribute to ferroptosis in neurons via lipid peroxidation [8]. Progressive systemic iron accumulation, mitochondrial dysfunction, and lipid peroxidation are associated with CIRI [9]. This pathological environment strongly links mitochondria and ferroptosis, suggesting that mitochondria play a pivotal role in CIRI through ferroptosis [10]. Although the interplay between mitochondrial homeostasis, inflammation, and ferroptosis in CIRI remains unclear, the inhibition of mitochondrial fission may be an effective strategy for CIRI recovery [11].

Considerable challenges remain in inhibiting mitochondrial fission in nerve cells. The blood‐brain barrier (BBB) prevents 98% of small and almost all large molecules from entering the brain, hindering the effectiveness of many neuroprotective drugs for CIRI treatment [12, 13, 14]. Neutrophils, which are short‐lived immune cells that migrate to the injury site after cerebral infarction, facilitate the trans‐BBB delivery of neutrophil‐targeting nanomaterials [15, 16, 17]. Although targeted neutrophil‐delivery drugs are promising for CIRI therapy, their similarity to other myeloid cells complicates selective targeting, increasing the risk of off‐target effects [18]. Additionally, the non‐selectivity of drugs at the ischemic site makes it difficult to target specific subcellular organelles, often leading to unsatisfactory efficacy and adverse side effects in clinical trials [19, 20].

**SCHEME 1:**
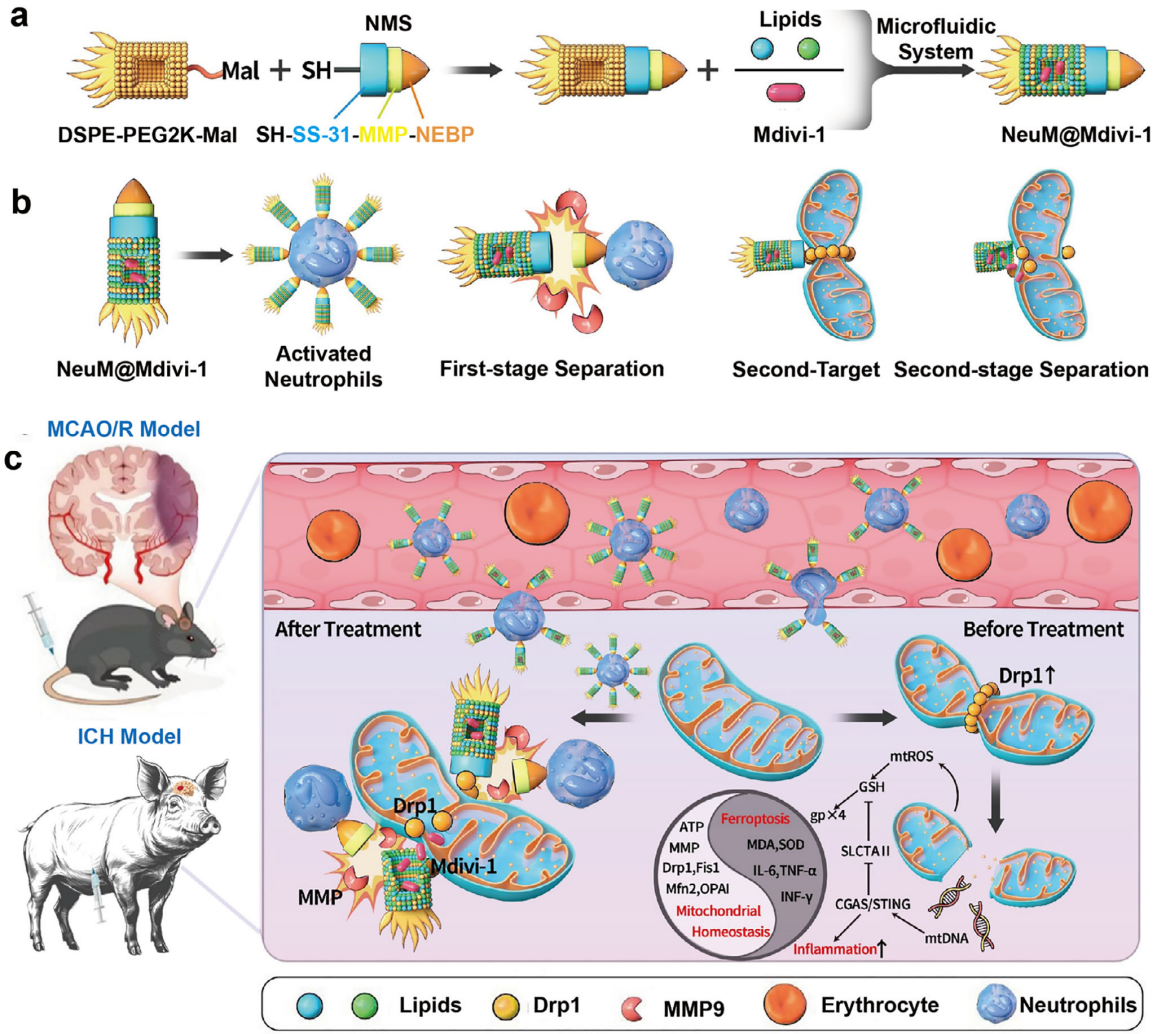
Schematic representation of constructing the rocket‐inspired NeuM@Mdivi‐1 nanoplatform and its mechanisms for CIRI therapy. NeuM@Mdivi‐1 sequentially targets neutrophils and mitochondria. (a) The preparation of NeuM@Mdivi‐1 lipid nanoparticles using a microfluidic system. (b) Schematic diagram of NeuM@Mdivi‐1 targeting and MMP9 responsiveness. (c) Activated neutrophils as natural carriers, NeuM@Mdivi‐1, administered via intravenous or intraperitoneal injection, traverse the blood‐brain barrier and target ischemic lesions. The nanoparticles are then released in response to the high MMP‐9 microenvironment, and a mitochondria‐targeting peptide facilitates the precise delivery of Mdivi‐1 to its site of action. This approach mediates its therapeutic effects against cerebral ischemia‐reperfusion injury through a triple mechanism: inhibiting pathological mitochondrial fission, attenuating inflammation, and counteracting ferroptosis. CIRI, cerebral ischemia‐reperfusion injury; ICH, intracerebral hemorrhage; MCAO/R, middle cerebral artery occlusion/reperfusion; MMP, matrix metalloproteinase; mtDNA, mitochondrial DNA.

We designed and constructed a matrix metalloproteinase‐9 (MMP9) responsive, two‐stage rocket‐inspired drug delivery platform to hierarchically deliver drugs in a spatiotemporal manner, target mitochondria undergoing excessive fission, and enhance brain‐targeted drug delivery in CIRI. Leveraging the elevated MMP9 levels at ischemic injury sites, we engineered a polypeptide (NMS) with MMP‐9 responsive cleavage capability, designed to target both neutrophils and mitochondria. This polypeptide was conjugated to DSPE‐PEG2K‐maleimide and self‐assembled with lipids and Mdivi‐1, a specific inhibitor of the mitochondrial protein Drp1, to form lipid nanoparticles (NeuM@Mdivi‐1) (Scheme 1A). Using the inherent inflammatory properties of neutrophils, these nanomaterials can effectively traverse the BBB and reach the injured site [21]. Through the enzymatic cleavage of engineered peptide linkers, the concealed SS‐31 peptide was released, thereby accomplishing the secondary delivery phase of the rocket‐inspired platform and enabling precise mitochondrial targeting through its inherent organelle‐homing capability. Using this dual‐stage approach aims to reduce mitochondrial division, prevent mtDNA release into the cytoplasm, inhibit the activation of the cGAS‐STING signaling pathway, and subsequently reverse neuroinflammation and iron‐induced cell death associated with CIRI. Ultimately, we aim to promote recovery from cerebral infarction. We highlight the therapeutic potential of a two‐stage rocket‐simulated drug delivery system for CIRI, offering a novel strategy to effectively address mitochondrial dysfunction and the associated inflammatory responses.

## 2 Results and Discussions

### 2.1 Design and Characterization of MMP9‐Responsive NeuM@Mdivi‐1

**FIGURE 1:**
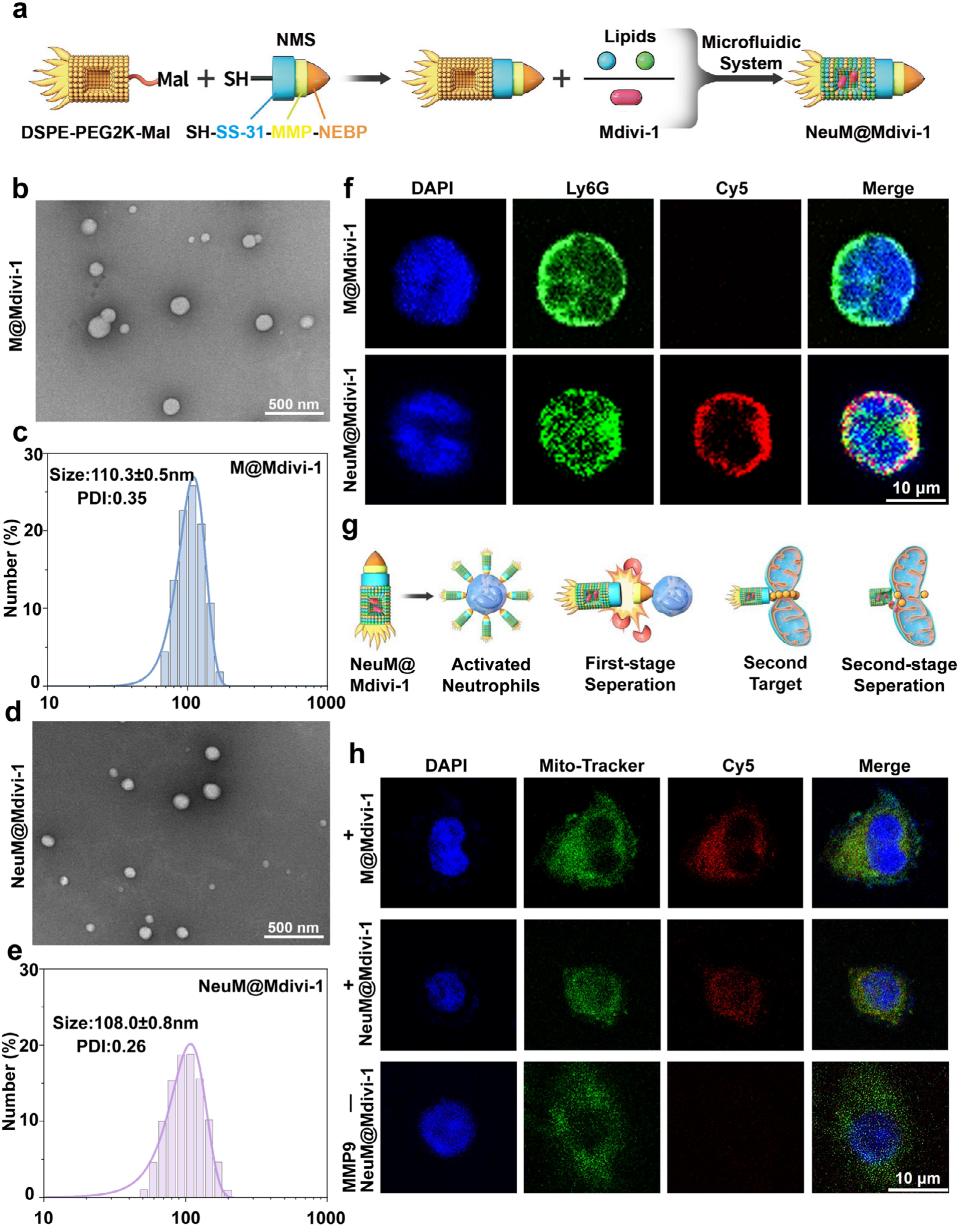
Synthesis and characterization of MMP9‐responsive neutrophil/mitochondrial‐targeted lipid nanoparticles. (a) Construction schematic diagram of NeuM@Mdivi‐1. (b) TEM images of M@Mdivi‐1 (scale bar = 500 nm). (c) The liposome size measured using DLS images of M@Mdivi‐1. (d) TEM images of NeuM@Mdivi‐1 (scale bar = 500 nm). (e) The liposome size measured using DLS images of NeuM@Mdivi‐1. (f) Validation of the ability of nanoparticles to target neutrophils (scale bar = 10 µm). (g) Schematic illustration of NeuM@Mdivi‐1 with MMP9 response. (h) Mitochondrial targeting ability of nanoparticles in PC12 cells (scale bar = 10 µm). TEM, transmission electron microscopy; DLS, dynamic light scattering.

The synthetic route for NeuM@Mdivi‐1 is shown in Figure 1a. The NEBP (targeting neutrophils; sequence: CGEAIPMSIPPEVK), MMP9‐responsive (sequence: PVGLIG), and SS‐31 (targeting mitochondria; sequence: H_2_N‐DArg‐Dmt‐Lys‐Phe‐NH_2_) peptides were conjugated to obtain the MMP9 responsive peptide NMS (sequence: DArg Dmt Lys (Cys)‐Phe‐PVGLIG‐GEAIPMSIPPEVK). The DSPE‐PEG‐NMS polymer was formed by modifying the polypeptide, NMS, on the surface of DSPE‐PEG2K‐maleimide raw material. Lipid nanoparticles, NeuM@Mdivi‐1 were prepared by combining the polymer with Mdivi‐1 and lipid molecules. As a control, mitochondria‐targeting lipid nanoparticles (M@Mdivi‐1) were prepared by using DSPE‐PEG2K‐SS31 instead of DSPE–PEG2K–NMS.

Characterization of the nanoparticles using complementary techniques, including transmission electron microscopy (TEM) and dynamic light scattering (DLS), revealed a uniform particle size distribution with an average diameter of approximately 110 nm (Figure 1b–e). Furthermore, the nanoparticles possessed had a zeta potential of approximately −28 mV (Figure S1), indicating a favorable surface charge that helps minimize non‐specific phagocytosis. To evaluate the membrane disruption toxicity, we conducted hemolysis using with red blood cells. Neither the assembled nanoparticles nor their dissociated components caused detectable hemolysis, which substantiated their excellent biocompatibility (Figure S2).

The targeting capabilities of the nanoparticles were evaluated across different cell types. Specifically, neutrophils were extracted from mouse bone marrow and activated with a prototypical *N*‐formylmethionine peptide (fMLP; 10 nm) for 60 min at 37°C. The activated neutrophils were subsequently exposed to nanoparticles for 6 h. Laser confocal microscopy revealed distinct fluorescence co‐localization between the Cy5‐labeled M@Mdivi‐1/ NeuM@Mdivi‐1 formulations and Ly6G‐positive neutrophil population, indicating efficient and specific neutrophil targeting by the nanomaterials (Figure 1f).

To validate the MMP9‐responsive mechanism of the nanoparticles, which relies on MMP9 overexpression to expose mitochondria‐targeting peptides, we investigated the effects of the nanoparticles on PC12 cells (Figure 1g). The cells were pre‐treated with or without 2 µm MMP9 enzyme and then incubated with the nanomedicine for 6 h. As visualized using laser confocal microscopy, the fluorescence co‐localization between the Cy5‐labeled drugs (M@Mdivi‐1 and NeuM@Mdivi‐1) and MitoTracker Green‐labeled mitochondria was observed exclusively in the presence of MMP9. These findings confirm that the mitochondria‐targeting capability of the nanomaterial is contingent upon MMP9 activation (Figure 1h).

Our drug delivery platform, inspired by the two‐stage rocket system used for satellite deployment, was designed to precisely transport different “drug satellites” to target cells and organelles. This system leverages endogenous neutrophils for targeted delivery to lesion sites, and incorporates MMP9‐responsive elements that enable spatiotemporally controlled drug release at the subcellular level. Unlike traditional MMP‐sensitive systems designed for tumor targeting, our platform overcomes the dependence on passive enhanced permeability and retention effects, achieving precise delivery of Mdivi‐1 to damaged mitochondria within the ischemic penumbra.

### 2.2 Validation of NeuM@Mdivi‐1 Targeting

**FIGURE 2:**
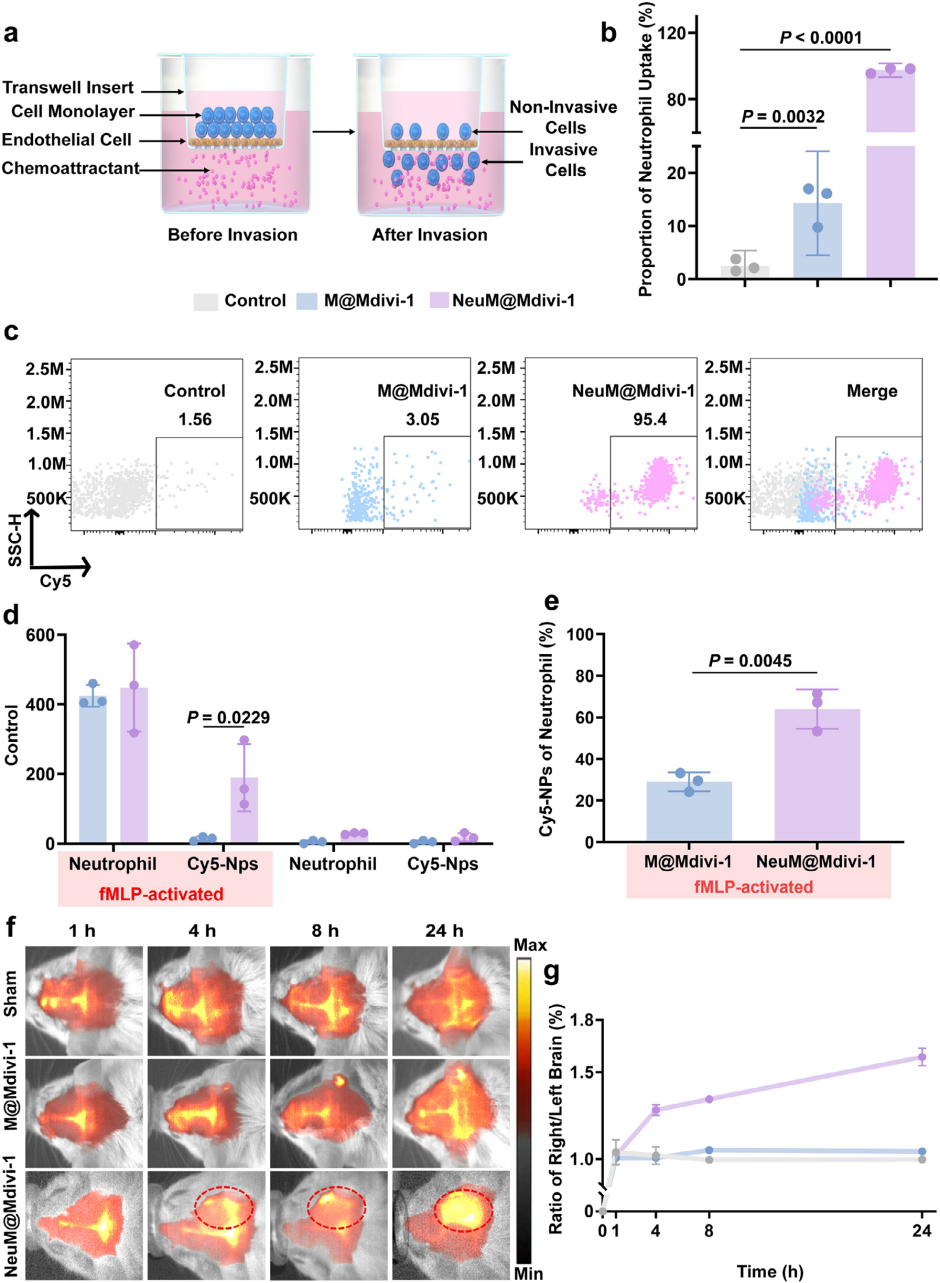
Neutrophil targeting ability and enhanced blood‐brain barrier penetration of lipid nanoparticles in ischemic stroke. (a) Transwell‐ simulated nanoparticles penetrate the blood‐brain barrier. (b) Quantification of the proportion of neutrophil uptake measured using flow cytometry; Data are presented as the mean ± SD. Statistical significance was analyzed using one‐way ANOVA followed by Tukey's multiple comparison test, and the *p* value marks the significance difference. *n* = 3. (c) Proportion of neutrophil uptake measured by flow cytometry; Statistical significance was analyzed using the Student's *t*‐test, *n* = 3. (d) Number of neutrophils penetrating endothelial cells; Statistical significance was analyzed using the Student's *t*‐test, *n* = 3 (e) Comparison of the targeting effect of Cy5‐labeled nanoparticles on activated neutrophils. (f) NIR II region imaging of mice brains, *n* = 3. (g) NIR II imaging quantification. The experiments were repeated three times, and representative data from one of the experiments are shown. fMLP, N‐formylmethionine peptide; NIR, Near‐InfraredSpectroscopy.

The transport of nanoparticles across the BBB was assessed using a Transwell assay, and neutrophil migration through the endothelial cell layers was quantified using flow cytometry (Figure 2a). Flow cytometry analysis revealed an obviously higher uptake rate of NeuM@Mdivi‐1 by activated neutrophils than that of M@Mdivi‐1 (Figure 2b,c). Moreover, results showed that activated neutrophils exhibited markedly greater penetration than unactivated cells (Figure 2d), and Cy5‐labeled NeuM@Mdivi‐1 demonstrated significantly greater BBB traversal efficiency than M@Mdivi‐1 (Figure 2e).

Based on the in vitro results, we evaluated the targeting efficacy of the nanoparticles in vivo. Acute ischemic stroke (IS) was induced in mice via middle cerebral artery occlusion (MCAO), a well‐established model [22]. Cy5‐labeled nanoparticles were intravenously administered, and its real‐time biodistribution in the brains of MCAO/reperfusion (MCAO/R) mice was monitored at designated time points (1, 4, 8, and 24 h) using NIR‐II imaging. The NeuM@Mdivi‐1 group exhibited a significantly stronger fluorescence signal in the brain than in that of the sham group at 24 h post‐injection (Figure 2f,g). Notably, the fluorescence intensity of NeuM@Mdivi‐1 in the ischemic hemisphere was markedly higher than that in the contralateral hemisphere, indicating selective distribution and specific targeting of NMS peptide‐modified liposomes to the infarcted area. Furthermore, ex vivo fluorescence imaging of the major organs was performed 24 h post‐injection to assess the overall biodistribution of NeuM@Mdivi‐1 (Figure S3). The signal pattern suggests that while nanoparticles can circulate to the lungs, those that fail to accumulate at the target site were subsequently cleared by the liver and kidneys.

### 2.3 NeuM@Mdivi‐1 Maintains Mitochondrial Homeostasis by Inhibiting Excessive Fission in CIRI

The brain has the highest oxygen consumption of any organ in the body and is highly vulnerable to hypoxic conditions [23]. It primarily relies on mitochondria within nerve cells to generate energy through the coordinated activity of the respiratory chain and ATP synthase. CIRI can induce severe mitochondrial dysfunction, manifesting as impaired energy metabolism and reduction in the mitochondrial membrane potential, which triggers oxidative stress and inflammatory responses [24]. To investigate the potential of nanoparticles to restore mitochondrial function, we established an in vitro anoxia‐reoxygenation model using PC12 cells and administered various drug treatments. Prior to the cellular experiments, the CCK‐8 assay was used to determine the appropriate concentration of the nanomedicine to treat PC12 cells. Of the concentrations used, cells remained viable at 10 µm, which was used for subsequent in vitro studies (Figure S4).

**FIGURE 3:**
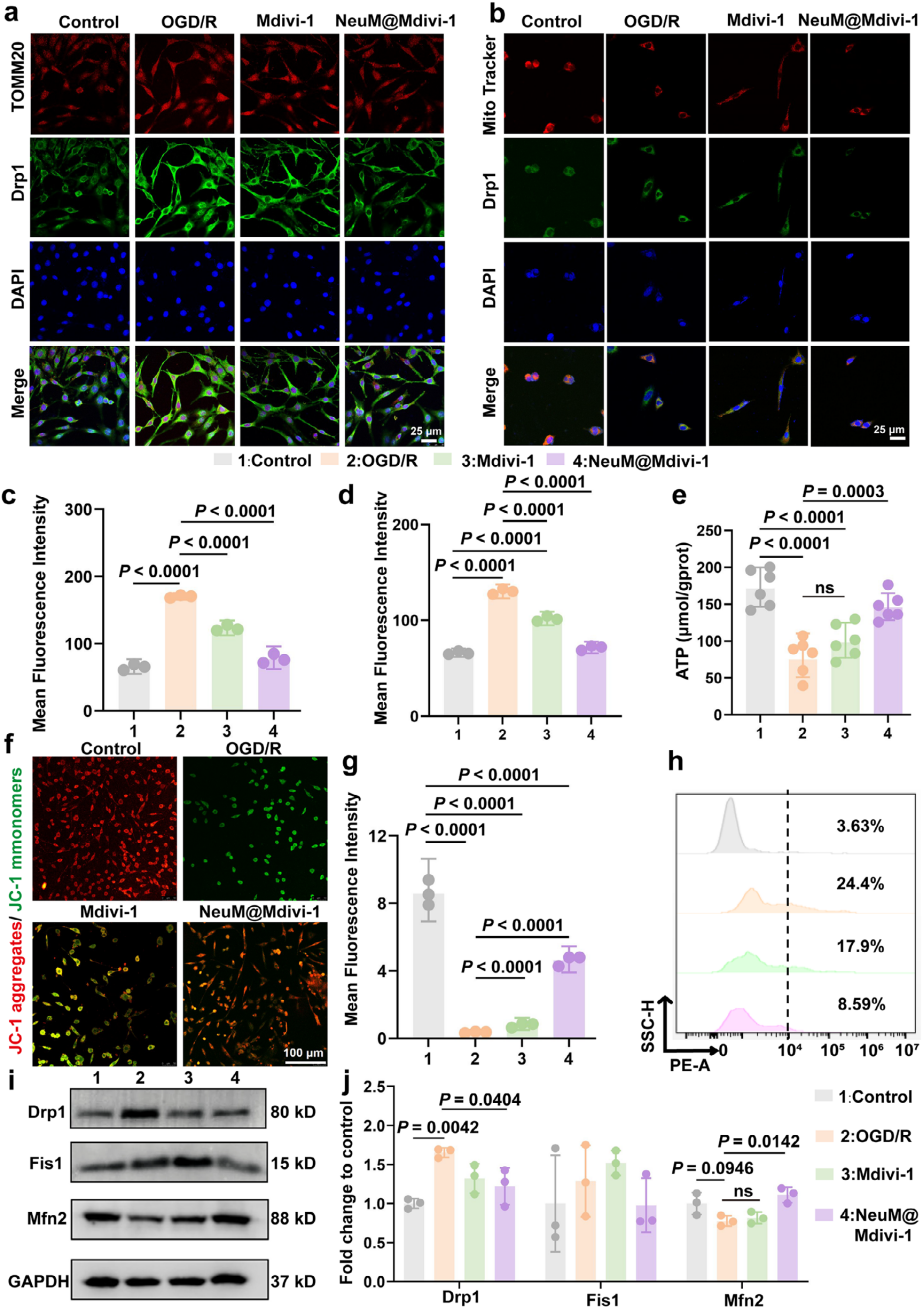
NeuM@Mdivi‐1 inhibits excessive mitochondrial division and maintains mitochondrial homeostasis. (a) Immunofluorescence co‐localization of Drp1 and TOMM20 (scale bar = 25 µm). (b) Fluorescence colocalization of mitochondrial and Drp1 probes (scale bar = 25 µm). (c) The quantitative analysis of Drp1 and TOMM20 co‐localization, *n* = 3. (d) The quantitative analysis of mitochondria and Drp1 co‐localization, *n* = 3. (e) The ATP content, *n* = 6. (f) MMP detection using a JC‐1 probe (scale bar = 100 µm). (g) The quantitative analysis of MMP, *n* = 3. (h) The mtROS levels in different groups. (i) Related protein expression levels of mitochondrial fission and fusion. (j) Related protein expression levels of mitochondrial fission and fusion. The experiments were repeated at least three times, and representative data from one of the experiments are shown. Data are presented as the mean ± SD. Statistical significance was analyzed using one‐way ANOVA using Tukey's post‐hoc and the *p*‐value marks the significant difference.

Under physiological conditions, mitochondria play a critical role in CIRI by maintaining a dynamic equilibrium through continual fusion and fission. Mitochondrial homeostasis regulates key processes such as energy metabolism, ROS generation, and apoptosis [25]. Our findings revealed an increase in mitochondrial fission following hypoxia‐reoxygenation, as evidenced by immunofluorescence co‐localization analysis, whereas treatment with NeuM@Mdivi‐1 reduced the fluorescence intensity of Drp1 and Fis1, indicating suppressed fission (Figure 3a–d). Moreover, the nanoparticles significantly upregulated the expression of the fusion‐related proteins Mfn2 and OPA1, and downregulated the fission‐related proteins Fis1 and Drp1 (Figure 3i,j). These results suggest that hypoxia‐reoxygenation disrupts the mitochondrial dynamic balance by promoting fission and impairing fusion, whereas NeuM@Mdivi‐1 counteracts this imbalance and contributes to restoring mitochondrial function.

The mitochondrial membrane potential is a key indicator of mitochondrial membrane integrity and functional status, and its decline is associated with mitochondrial dysfunction and inadequate ATP synthesis [26]. During ATP production, mitochondria generate ROS as byproducts of the electron transport chain located in the inner mitochondrial membrane. Excessive ROS accumulation impairs mitochondrial function and promotes autophagy and apoptosis [27]. Therefore, we assessed the mitochondrial membrane potential, ATP content, and mtROS levels following different treatments. After CIRI, the ATP content (Figure 3e) and MMP levels (Figure 3f,g) markedly decreased, and mtROS levels significantly increased (Figure 3h). However, this effect was reversed by treatment with NeuM@Mdivi‐1. These findings indicate that NeuM@Mdivi‐1 effectively restored mitochondrial function after hypoxia‐reoxygenation injury.

### 2.4 NeuM@Mdivi‐1 Reverses Neuroinflammation Through the mtDNA/cGAS‐STING Axis and Inhibits Ferroptosis

**FIGURE 4:**
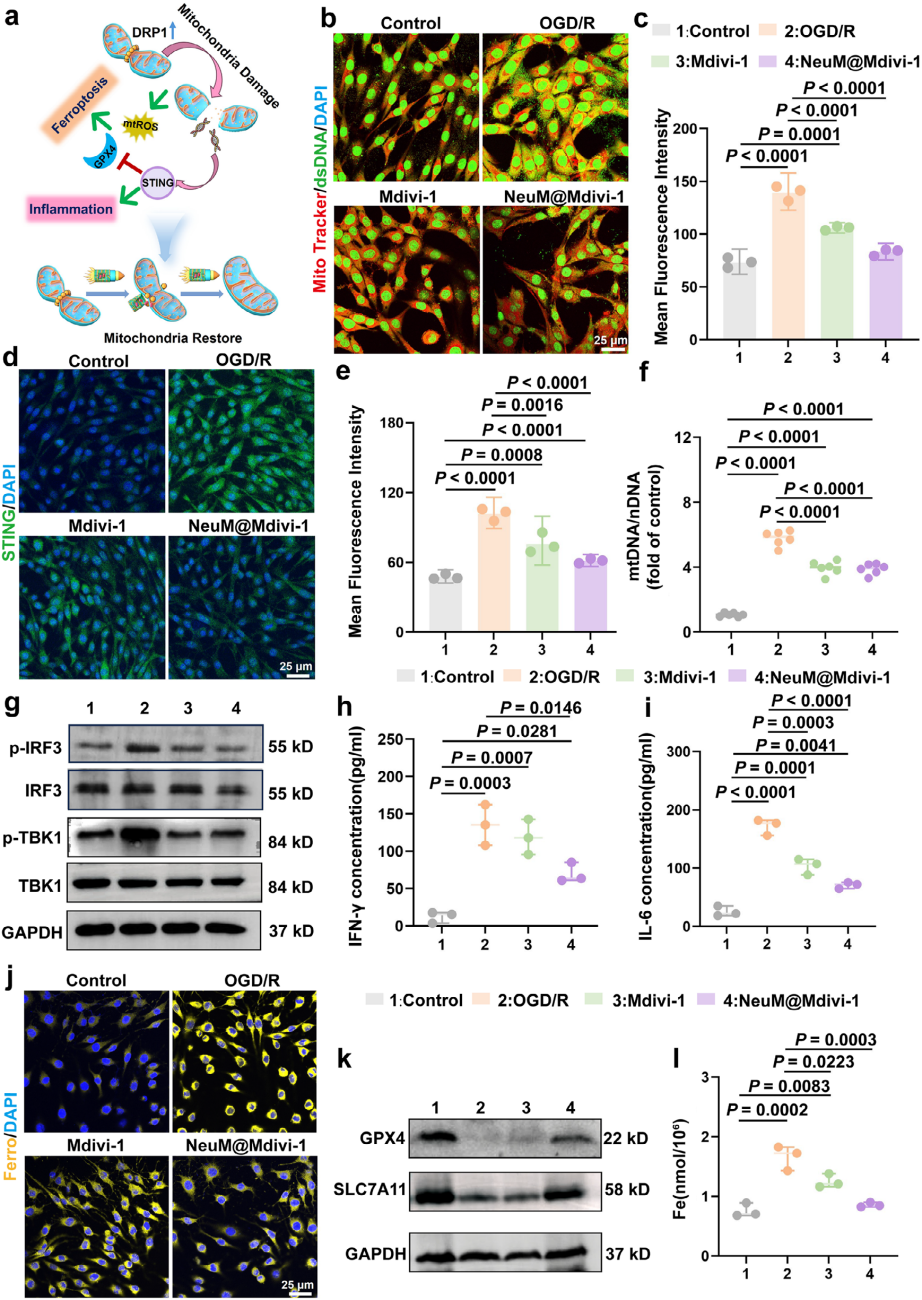
NeuM@Mdivi‐1 reduces inflammatory responses and inhibits ferroptosis. (a) Schematic diagram of the mechanism by which NeuM@Mdivi‐1 inhibits DRP1 to rescue excessively fragmented mitochondria and restore mitochondrial homeostasis. (b) Fluorescence colocalization of mitochondrial and dsDNA (scale bar = 25 µm). (c) The quantitative analysis of mitochondrial and dsDNA co‐localization, *n* = 3. (d) The fluorescence expression of STING (scale bar = 25 µm). (e) The quantitative analysis of STING, *n* = 3. (f) The mtDNA copy number, *n* = 6. (g) Related protein expression levels of STING pathway. (h) The content of IFN‐γ, *n* = 3. (i) The content of IL‐6 in different groups, *n* = 3. (j) The fluorescence expression of Fe^2+^ (scale bar = 25 µm). (k) Related protein expression levels of ferroptosis. (l) The Fe^2+^ content. The experiments were repeated at least three times and representative data from one of the experiments are shown. Data are presented as the mean ± SD. Statistical significance was analyzed using one‐way ANOVA using Tukey's post‐hoc and the *p* value marks the significant difference. mtDNA, mitochondrial DNA.

Mitochondrial damage following ischemia‐reperfusion injury facilitates the release of mtDNA into the cytoplasm, along with an increase in mitochondrial fragments [28]. This leads to the cytosolic accumulation of mtDNA and subsequent activation of the cGAS‐STING signaling pathway, which promotes type I interferons (IFN) and inflammatory cytokines, thereby exacerbating CIRI (Figure 4a) [29]. To investigate the inflammatory response under these conditions, we established an in vitro hypoxia‐reoxygenation model using PC12 cells. After the NeuM@Mdivi‐1 treatment, the fluorescence spots of dsDNA (Figure 4b,c) and STING (Figure 4d) significantly decreased, and the copy number of mtDNA significantly decreased (Figure 4f). Furthermore, we examined the expression of key proteins in the cGAS/STING pathway (—IRF3, and TBK1). NeuM@Mdivi‐1 markedly suppressed IRF3 and TBK1 phosphorylation (Figure 4g, Figure S5). The levels of pro‐inflammatory cytokines, IL‐6 (Figure 4h), IFN‐γ (Figure 4i), and TNF‐α (Figure S6) were significantly reduced. Collectively, these findings indicate that NeuM@Mdivi‐1 attenuates the inflammatory response associated with CIRI.

During CIRI, tissue damage leads to an increased release of iron ions within cells. During ferroptosis, the intracellular redox balance is disrupted, glutathione peroxidase 4 (GPX4) activity is inhibited, and the capacity to scavenge excess ROS is diminished, thereby promoting ferroptosis [30]. We applied iron ion probes to observe the levels of iron ions in cells after hypoxic reoxygenation. The fluorescence intensity of iron ions in the oxygen and glucose deprivation (OGD) group significantly increased, whereas it decreased after treatment with NeuM@Mdivi‐1 (Figure 4j, Figure S7). The expression of key proteins involved in the ferroptosis pathway, GPX4 and SLC7A11 was significantly up‐regulated (Figure 4k, Figure S8). Furthermore, we measured the levels of Fe^2^^+^, glutathione (GSH), and malondialdehyde (MDA) across different treatment groups. NeuM@Mdivi‐1 effectively reduced Fe^2^^+^ (Figure 4i) and MDA (Figure S9a) levels, and elevated GSH levels (Figure S9b). Overall, these results indicate that NeuM@Mdivi‐1 alleviates inflammation and oxidative stress following hypoxia‐reoxygenation injury, and inhibits ferroptosis, thereby mitigating CIRI.

### 2.5 Evaluation of the Therapeutic Effect and In Vivo Biological Safety of NeuM@Mdivi‐1

**FIGURE 5:**
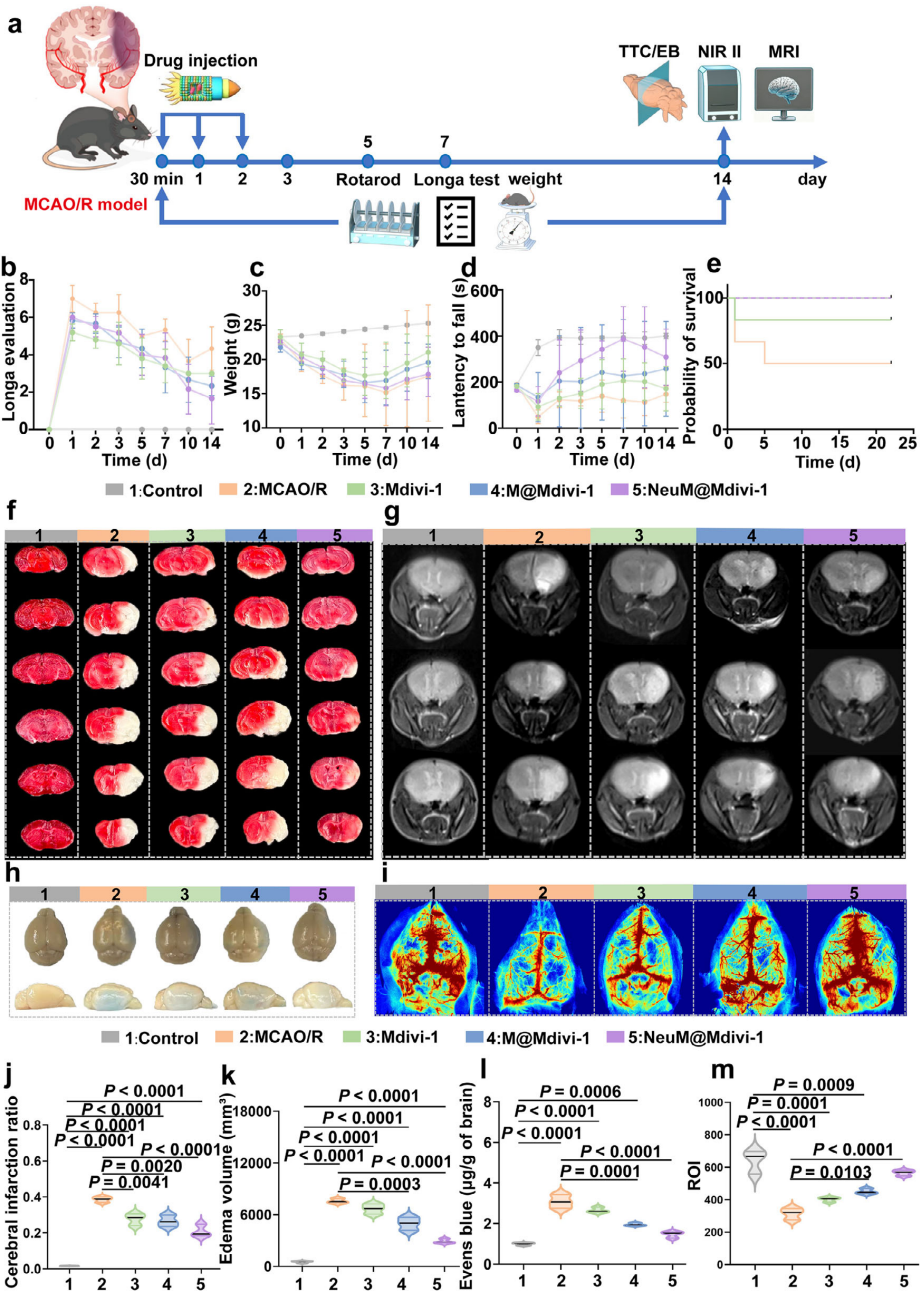
Nanoparticles ameliorated long‐term neurological deficits and alleviated BBB disruption and ischemia‐reperfusion injury in MCAO/R mice. (a) Schematic image of the timeline of the MCAO/R model receiving nanoparticle therapy. (b) The rotarod test was statistically analyzed, *n* = 4–6 per group. (c) The changes in body weight in different treatment groups after MCAO/R in mice, *n* = 4–6 per group. (d) Longa scores in the different groups, *n* = 4–6 per group. (e) Survival curve, *n* = 3. (f) Representative TTC staining images of the infarct volume of brain slices treated with different liposomes. (g) MRI T2‐weighted imaging was used to evaluate cerebral edema (h) EB staining was used to detect the degree of damage to the BBB. (i) Brain laser speckle imaging. (j) Quantitative analysis of the infarct volume from TTC, *n* = 3. (k) Quantitative analysis of the brain edema calculated using MRI, *n* = 3. (l) Quantitative analysis of the concentration of EB in brain tissue, *n* = 3. (m) Quantitative analysis of laser speckle imaging in the brain tissue, *n* = 3. Data are presented as the mean ± SD. Statistical significance was analyzed using one‐way ANOVA followed by Tukey's multiple comparison test and the *p* value marks the significant difference, *n* = 3. BBB, blood‐brain barrier; EB, Evan's Blue; MCAO/R, middle cerebral artery occlusion/reperfusion.

To evaluate the therapeutic potential of NeuM@Mdivi‐1 in CIRI, we investigated its efficacy in an MCAO/R mouse model (Figure 5a). At 30 min after reperfusion, the mice were randomly assigned to treatment groups and administered PBS, Mdivi‐1, M@Mdivi‐1, or NeuM@Mdivi‐1 (1 mg/kg) via tail vein injection, with continued dosing for 3 days. Body weight was monitored to assess the protective effect of the treatments on the central nervous system. A marked reduction in body weight was observed in all groups on the first day after MCAO/R surgery. Neurological function, a key indicator for assessing IS treatment, was evaluated using the Longa score and rotarod performance on post‐reperfusion days 1, 2, 3, 5, 7, and 14. The MCAO/R group displayed severe neurological deficits, characterized by flexion and sustained circling of the right forelimb. The drug treatment promoted functional recovery and partially ameliorated the hemiplegic symptoms. Notably, the NeuM@Mdivi‐1 group exhibited a more pronounced reduction in neurological deficits than that of the other treatment groups (Figure 5b). Weight recovery occurred after drug intervention, with the NeuM@Mdivi‐1 group exhibiting the most rapid restoration, suggesting improved therapeutic outcomes in MCAO/R mice (Figure 5c). Rotarod testing revealed a significant decline in the performance of MCAO/R mice relative to that of the sham group. All treatment groups exhibited improved rotarod performance, with NeuM@Mdivi‐1 demonstrating the most substantial effect (Figure 5d). Moreover, the survival rate of MCAO/R mice was markedly improved by NeuM@Mdivi‐1 treatment compared with that of mice receiving PBS, Mdivi‐1, or M@Mdivi‐1, further supporting its protective role against CIRI (Figure 5e).

To evaluate the protective effect of the nanoparticles on the ischemic penumbra, TTC staining was performed 72 h post‐surgery. A substantial infarct area (38.6%) was observed in the MCAO/R group (Figure 5f,j), confirming the successful establishment of the model. In contrast, the infarct sizes in the treatment groups were 27.7%, 26.5%, and 20.9%, respectively. Notably, the NeuM@Mdivi‐1 group exhibited the most significant reduction in the infarct size, suggesting effective targeting of the ischemic region. Quantitative analysis of the magnetic resonance imaging (MRI) results at 72 h post‐reperfusion further corroborated this finding, showing a minimal infarct volume in the NeuM@Mdivi‐1 group (Figure 5g,k), underscoring its pronounced therapeutic efficacy. Furthermore, the BBB integrity is critical for mitigating secondary brain injury after acute IS, because the BBB disruption considerably increases the risk of hemorrhagic transformation following revascularization, which is a key factor associated with poor patient prognosis. We assessed the BBB permeability using the Evans Blue (EB) leakage assay. The BBB permeability markedly increased in the ischemic hemisphere of MCAO/R mice compared with that in the sham group (Figure 5h,l), as evidenced by extensive EB extravasation and accumulation. In contrast, the BBB integrity was significantly preserved with NeuM@Mdivi‐1 treatment. Additionally, we conducted laser speckle imaging to observe vascular repair after ischemia‐reperfusion injury. The NeuM@Mdivi‐1 group exhibited vascular regeneration and restored blood flow in the infarcted area (Figure 5i,m). Additionally, serum and various organ tissues were collected to evaluate the potential systemic toxicity. Assessments of liver and kidney functions, along with the Hematoxylin‐Eosin (HE) staining of major organs, indicated no significant nanomaterial‐associated toxic effects (Figures S10 and S11). Collectively, these results highlight the considerable therapeutic potential of NeuM@Mdivi‐1 for treating of CIRI.

### 2.6 NeuM@Mdivi‐1 Restores Mitochondrial Homeostasis and Attenuates Inflammation and Ferroptosis in Cerebral Ischemia

To elucidate the effects of the nanoparticles on mitochondrial dynamics and neuroinflammation, we conducted comprehensive analyses of brain tissue from MCAO/R mice.

**FIGURE 6:**
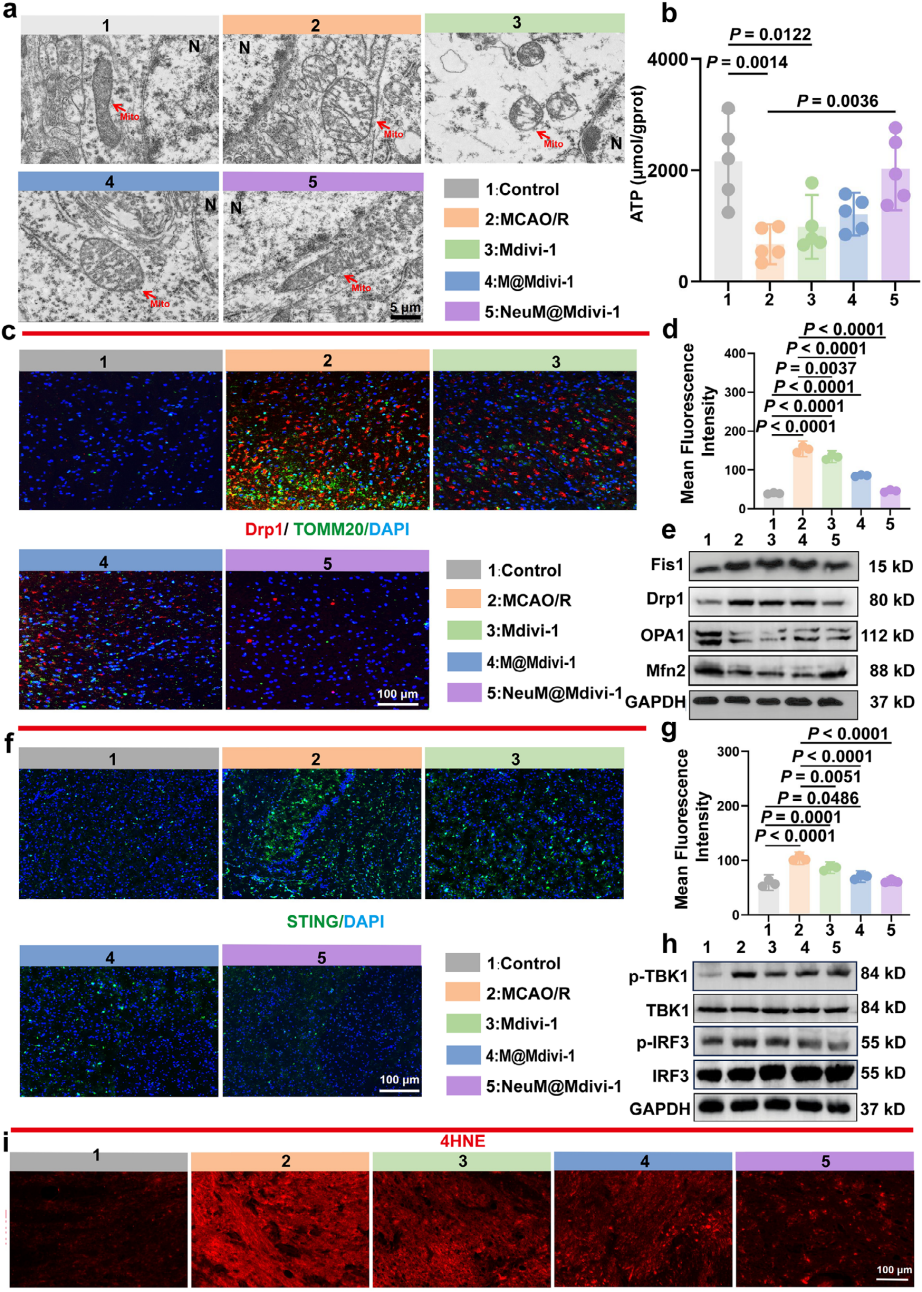
The effects of nanoparticles on mitochondrial dynamics, inflammatory responses and ferroptosis. (a) Ultrastructure of the brain tissue. Red arrows indicate mitochondrion. “N” means nucleus (scale bar = 5 µm). (b) The ATP content, *n* = 5. (c) Immunofluorescence colocalization of Drp1 and TOMM20 in brain tissue (scale bar = 100 µm). (d) The quantitative analysis of Drp1 and TOMM20 co‐localization, *n* = 3. (e) Related protein expression levels of mitochondrial dynamics, (n = 3 mice in each group). (f) Immunofluorescence of STING in brain tissue (scale bar = 100 µm). (g) The quantitative analysis of STING, *n* = 3. (h) Related protein expression levels of the cGAS‐STING pathway, (n = 3 mice in each group). (i) Immunofluorescence of 4HNE in brain tissue (scale bar = 100 µm). Data are shown as the mean ± SD. Representative data from one of the experiments are shown. Statistical significance was analyzed using one‐way ANOVA followed by Tukey's multiple comparison test and the *p* value marks the significant difference. MCAO/R, middle cerebral artery occlusion/reperfusion.

Transmission electron microscopy (TEM) revealed severe ultrastructural damage to mitochondria in the MCAO/R group, characterized by cristae rupture, vacuolization, and shortened mitochondrial length due to fission (Figure 6a). After the drug treatment, the morphology of the mitochondria in each treatment group showed some recovery, with significantly decreased mitochondrial swelling and vacuolization, and increased mitochondrial length in the M@Mdivi‐1 and NeuM@Mdivi‐1 groups. Additionally, the ATP content in the brain was measured to evaluate the recovery of mitochondrial function. The ATP content significantly increased in the NeuM@Mdivi‐1 group (Figure 6b). Immunofluorescence co‐staining results of Drp1 and TOMM20 in ischemic brain tissue demonstrated enhanced co‐localization following ischemia‐reperfusion, indicating elevated mitochondrial fission (Figure 6c,d). This effect was reversed in the treatment groups, with the NeuM@Mdivi‐1 group showing the most pronounced effect. The western blot analysis confirmed that the NeuM@Mdivi‐1 treatment significantly suppressed mitochondrial fission and promoted mitochondrial fusion (Figure 6e, Figure S12). Additionally, the mtDNA copy number was notably reduced in the NeuM@Mdivi‐1 group compared with that in the MCAO/R group (Figure S13), suggesting attenuated mtDNA release. These in vivo findings are consistent with our in vitro results, indicating that NeuM@Mdivi‐1 mitigates pathological mitochondrial fission, limits mtDNA release, and restores mitochondrial function and homeostasis.

We assessed the levels of key factors associated with the cGAS–STING pathway in the brain tissue. The nanoparticles markedly decreased the fluorescence expression of STING (Figure 6f,g), p‐TBK1, and p‐IRF3 protein expression levels in the brain (Figure 6h, Figure S14). Additionally, the concentrations of TNF‐α, IL‐6, and IFN‐γ (Figure S15) were significantly reduced. These results indicated that the nanoparticles attenuate neuroinflammation by suppressing the cGAS–STING pathway.

Excess iron ions can produce a large number of ROS through the Fenton reaction, which specifically reacts with polyunsaturated fatty acids in cell membranes, causing lipid peroxidation, destroying the structure and function of cell membranes, and leading to cell rupture and death [31]. Under oxidative stress or other damaging conditions, lipid peroxidation leads to the generation of 4‐hydroxynonenal (4‐HNE) from fatty acids. Immunofluorescence analysis of the brain tissue revealed that NeuM@Mdivi‐1 significantly reduced the fluorescence puncta of 4‐HNE (Figure 6i, Figure S16a). The intracellular redox balance disruption, GPX4 activity inhibition, and diminished capacity to scavenge excess ROS collectively promote ferroptosis [32]. In this study, we demonstrated that NeuM@Mdivi‐1 treatment significantly increased GPX4 and SLC7A11 protein expression (Figure S16b,c), and GSH levels (Figure S16d), while decreasing Fe^2+^ (Figure S16e) and MDA (Figure S16f) levels in the brain of MCAO/R mice, thereby effectively suppressing ferroptosis in CIRI. The notable therapeutic efficacy of NeuM@Mdivi‐1 was primarily due to its multi‐target actions on the key pathological cascades involved in CIRI progression. By precisely targeting damaged mitochondria, NeuM@Mdivi‐1 acts at upstream regulatory nodes in the mitochondrial dysfunction cascade, mitigating oxidative damage and inflammatory amplification, while restoring mitochondrial homeostasis. Consequently, NeuM@Mdivi‐1 not only offers a promising therapeutic strategy for CIRI, but also has potential for the treatment of other ischemic diseases.

### 2.7 NeuM@Mdivi‐1 Mitigates Intracerebral Hemorrhaging in Porcine Models

To evaluate the cross‐species therapeutic potential of our nano‐delivery platform, we used a porcine intracerebral hemorrhage (ICH) model, a physiologically relevant large animal system for stroke research [33]. NeuM@Mdivi‐1 (5 mg/kg) was intraperitoneally administered 30 min after ICH induction, followed by repeated dosing every 24 h. Neurological recovery, imaging biomarkers, and inflammatory responses were systematically assessed over a 7d period.

**FIGURE 7:**
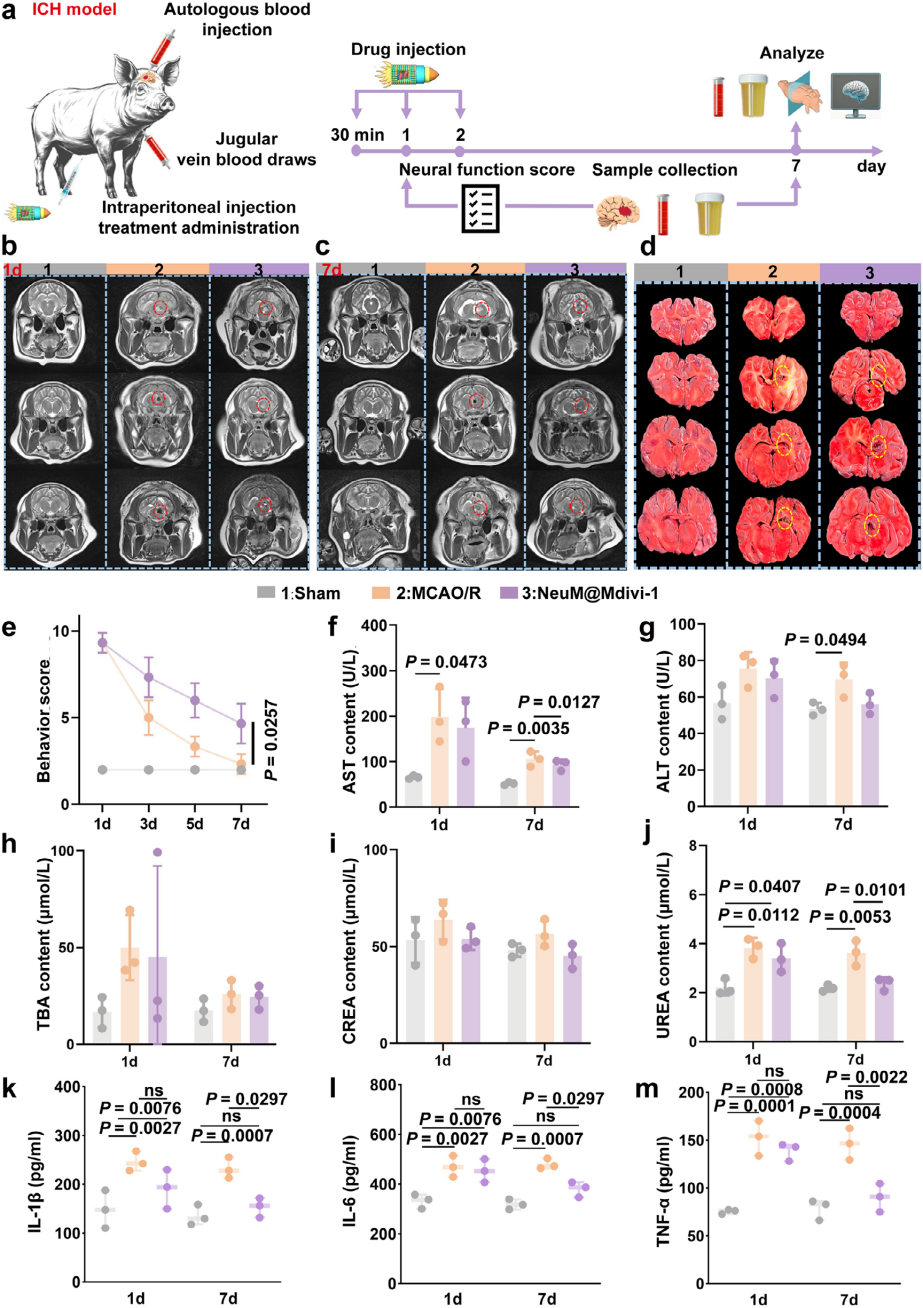
NeuM@Mdivi‐1 ‐mediated reduction of hematomas and inflammation in the ICH porcine model. (a) Timeline of the ICH piglets receiving the nanoparticle treatment. (b) MRI T2‐weighted imaging was used to evaluate cerebral edema on day 1. The red dotted circle is the site of the hematoma. (c) MRI T2‐weighted imaging was used to evaluate the cerebral edema on day 7. The red dotted circle is the site of the hematoma. (d) Representative TTC staining images of the infarct volume of brain slices treated with different liposomes. (e) Purdy neural function score, *n* = 3 per group. (f) The AST content, *n* = 3. (g) The ALT content, *n* = 3. (h) The TBA content, *n* = 3. (i) The CREA content, *n* = 3. (j) The UREA content, *n* = 3. (k) The IL‐1β content in cerebrospinal fluid, *n* = 3. (l) The IL‐6 content in cerebrospinal fluid, *n* = 3. (m) The TNF‐α content in cerebrospinal fluid, *n* = 3. Data are shown as the mean ± SD. Representative data from one of the experiments are shown. Statistical significance was analyzed using one‐way ANOVA followed by Tukey's post‐hoc test, and the *p* value marks the significant difference. ICH, intracerebral hemorrhage; MCAO/R, middle cerebral artery occlusion/reperfusion.

On post–ICH days 1 and 7 TTC staining, MRI, inflammatory marker analysis in the cerebrospinal fluid, and serum biochemical profiling were conducted. Behavioral assessments and neurological function evaluations were performed on days 1, 3, 5, and 7 (Figure 7a). All experimental subjects survived until the predetermined endpoint on day 7.

MRI was conducted on post‐ICH day 1. Distinct hematoma regions were observed in both the ICH and NeuM@Mdivi‐1 groups, confirming the successful establishment of the model (Figure 7b). On day 7, the follow‐up MRI of the same animals showed a marked reduction in hematoma volume in the NeuM@Mdivi‐1 group, whereas no significant improvement was observed in the ICH group (Figure 7c). Consistently, TTC staining of brain tissues harvested on day 7 showed notably delineated infarct areas in the ICH model group, which were notably absent in the NeuM@Mdivi‐1 group (Figure 7d).

Following the induction of ICH in pigs, neurological impairment was the most severe on post–ICH day 1. The affected animals remained conscious but were unable to stand and exhibited head turning on one side, reduced appetite, and hypersalivation. Neurological scores progressively improved over time in the NeuM@Mdivi‐1 group and were significantly lower than those in the ICH group on post‐ICH days 3, 5, and 7 (Figure 7e), indicating enhanced functional recovery.

Liver and kidney function indices were assessed. Except for the significant increases in AST (Figure 7f), ALT (Figure 7g), and UREA (Figure 7j) levels observed in the ICH group during the experiment, no notable differences were detected in TBA (Figure 7h), or CREA (Figure 7i). These findings suggest that NeuM@Mdivi‐1 promotes recovery from ICH–induced brain injury in pigs, attenuates neuroinflammation, and does not exhibit significant systemic toxicity.

During the experimental period, inflammatory factors in cerebrospinal fluid were quantified. On the post–ICH day 1, the levels of IL‐1β (Figure 7k), IL‐6 (Figure 7l), and TNF‐α (Figure 7m) were significantly elevated in both the ICH and NeuM@Mdivi‐1 groups compared with those in the sham group. However, on the post–ICH day 7, the NeuM@Mdivi‐1 group exhibited a marked reduction in the concentrations of these cytokines compared with those of the ICH group.

Collectively, our study confirms the efficacy of nanomedicine not only in rodent MCAO/R models, but also, and more notably, in the translational ICH porcine model. This cross‐species validation represents a valuable step forward in neurological nanomedicine, demonstrating therapeutic potential in a large‐animal setting, an advancement that is rarely achieved in the field.

### 2.8 Impact of NeuM@Mdivi‐1 on Bioinformatics

**FIGURE 8:**
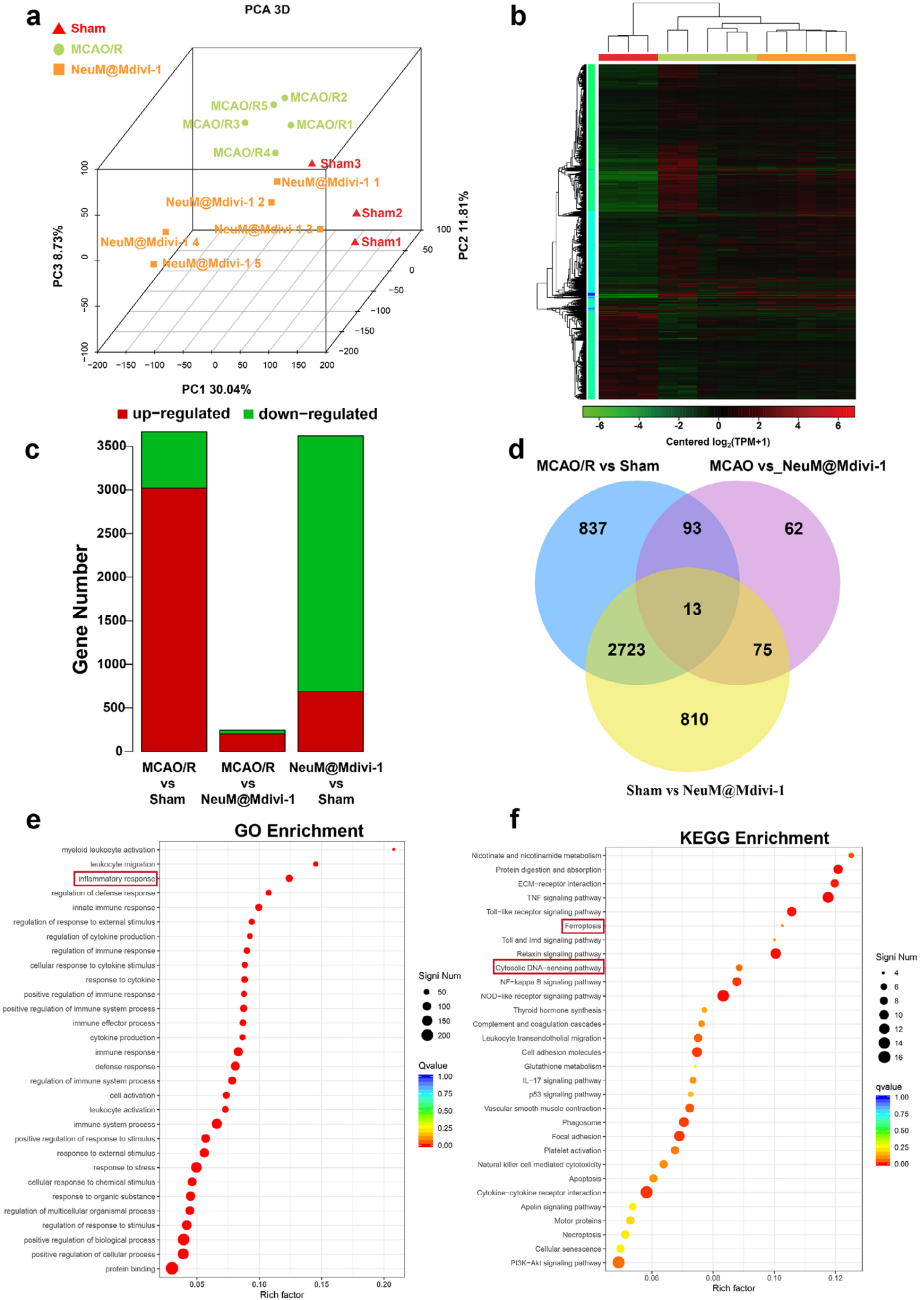
Identification of differentially expressed genes. (a) PCA of normalized RNA‐seq data matrix of Sham, MCAO/R, and NeuM@Mdivi‐1 groups. (b) Heatmap showing differentially expressed genes in Sham, MCAO/R, and NeuM@Mdivi‐1. (c) Analysis of expression differences: statistical bar chart. (d) VENN diagram of expressed genes in the treatment groups. (e) The GO analysis of differential gene expression between different treatment groups. (f) The KEGG analysis of differential genes in the treatment groups. GO, Gene Ontology; KEGG, Kyoto Encyclopedia of Genes and Genomes; MCAO/R, middle cerebral artery occlusion/reperfusion; PCA, principal component analysis.

To elucidate the potential mechanisms underlying the neuroprotective effects of NeuM@Mdivi‐1, RNA sequencing (RNA‐seq) was performed on brain tissues from three experimental groups: sham surgery, MCAO/R, and NeuM@Mdivi‐1‐treated mice. Multivariate principal component analysis (PCA) revealed clear segregation among the three groups, with principal components 1 (30.04% variance), 2 (11.81% variance), and 3 (8.73% variance) collectively capturing the major transcriptional variations (Figure 8a). Hierarchical clustering of gene sets further illustrated distinct expression profiles across groups (Figure 8b). The numbers of upregulated and downregulated differentially expressed genes (DEGs) markedly differed between the groups (Figure 8c). A Venn diagram further delineated the overlap and uniqueness of the gene expression patterns among the experimental conditions (Figure 8d). Specifically, compared with the sham group, the MCAO/R group exhibited 3666 DEGs, including 3020 upregulated and 646 downregulated genes. In contrast, the NeuM@Mdivi‐1–treated group exhibited 243 DEGs compared with those of the MCAO/R group, of which 201 were upregulated and 42 were downregulated.

Gene Ontology (GO) and Kyoto Encyclopedia of Genes and Genomes (KEGG) enrichment analyses were performed using false discovery rate (FDR)‐adjusted values (Benjamini–Hochberg correction, Q < 0.05). The nanoparticles significantly modulated multiple signaling pathways associated with inflammatory responses, ferroptosis, mitochondrial homeostasis, and oxidative stress (Figure 8e,f). GO analysis revealed that the DEGs between the NeuM@Mdivi‐1 and MCAO/R groups were predominantly enriched in inflammatory and immune response pathways. This is consistent with our experimental findings that NeuM@Mdivi‐1 alleviates CIRI by reducing pro‐inflammatory cytokines such as TNF‐α and IL‐6, and interfering with the cGAS‐STING signaling axis—a central pathway in immune activation. Furthermore, KEGG pathway analysis showed significant enrichment in ferroptosis and cytosolic, DNA‐sensing pathways. This aligns with our mechanistic evidence that NeuM@Mdivi‐1 maintains mitochondrial homeostasis, thereby reducing mtDNA release, ROS production, and lipid peroxidation, and consequently suppressing CIRI–induced ferroptosis. However, these findings remain preliminary and require further in vitro and in vivo validation to confirm the involvement of these pathways and to elucidate their specific contributions to the observed therapeutic effects. In summary, NeuM@Mdivi‐1 exerts its therapeutic effects in IS by maintaining mitochondrial homeostasis and suppressing inflammation and ferroptosis, thereby offering an efficient and innovative strategy for treating IS.

Our study had several limitations. Although we used different species and animal models to enhance the generalizability of our findings, the relatively small sample size may have limited the reliability of the results. Additionally, the nanoplatform requires the optimization of clinically relevant parameters, including dosage, timing, sequence, administration routes, and in vivo metabolic pathways, with long‐term assessment of biosafety in practical applications. Furthermore, although consistent peptide mass ratios were maintained to evaluate the role of NMS, the optimal peptide concentration for BBB penetration requires further investigation. Based on these limitations, future research should include: developing more clinically representative models to better simulate human pathophysiology, conducting in‐depth investigations on the pharmacokinetics and pharmacodynamics of nanoparticles in vivo, and optimizing administration protocols. This study provides new strategies and insights for the treatment of IS. Future research building on this work may contribute to the development of new theoretical frameworks and practical approaches for treating IS and other brain disorders.

## 3 Conclusions

In summary, we designed a novel drug delivery platform that simulates a two‐stage rocket to deliver therapeutic “satellites.” It can sequentially target neutrophils and mitochondria, overcoming the limitations of drugs that have difficulty passing through the BBB and accurately targeting subcellular structures. It can effectively reduce mitochondrial fission while maintaining dynamics and protecting mitochondrial function. More importantly, it reduces mtDNA release, inhibits activation of the cGAS–STING signaling pathway, attenuates neuroinflammation and ferroptosis, and improves CIRI.

## 4 Methods

### 4.1 Ethical Compliance

All procedures involving animals were performed in strict adherence to the Guidelines for the Management of Laboratory Animals promulgated by the State Science and Technology Commission of China. The study protocols were reviewed and approved by the Animal Welfare and Ethics Committees of Mudanjiang Medical University and Northeast Agricultural University (Approval Nos. IACUC‐20230806‐136 and NEAIEC202403206).

### 4.2 Synthesis of M@Mdivi‐1 and NeuM@Mdivi‐1

A neutrophil‐targeting peptide NEBP (CGEAIPMSIPPEVK), an MMP9‐cleavable peptide (PVGLIG), and the mitochondria‐directing peptide SS‐31 (H_2_N‐DArg‐Dmt‐Lys‐Phe‐NH_2_) were conjugated at an equimolar ratio to form the multifunctional peptide NMS (DArg‐Dmt‐Lys (Cys)‐Phe‐PVGLIG‐GEAIPMSIPPEVK). This peptide was then coupled to DSPE‐PEG2K_−_Maleimide at a 1:1 molar ratio. The resulting conjugate was combined with Mdivi‐1 and the fluorescent tracer Cy5 at a molar ratio of 5:1:0.2, dissolved in dichloromethane, and formed into a thin film by rotary evaporation. The film was hydrated with phosphate‐buffered saline (PBS) and sonicated for 10 min to obtain NeuM@Mdivi‐1. For comparison, non‐neutrophil‐targeted M@Mdivi‐1 was fabricated using an analogous procedure.

### 4.3 Oxygen‐Glucose Deprivation/ Reperfusion (OGD/R) Model

PC12 cells (CL‐0412, Wuhan Pricella Biotechnology) were first cultured for 24 h under standard conditions. The culture medium was then replaced with glucose‐ and serum‐free DMEM (Gibco), and cells were transferred to a tri‐gas incubator with an atmosphere of 95% N_2_ and 5% CO_2_ to induce hypoxic injury. After 4 h of oxygen–glucose deprivation, the medium was changed back to complete medium, and cells were returned to a normoxic incubator to simulate reperfusion for 24 h.

### 4.4 Isolation of Neutrophils from Murine Bone Marrow

Femurs and tibias were dissected from mice, and bone marrow cells were flushed out using a homogenate rinsing solution (480058). Neutrophils were isolated via density gradient centrifugation according to the manufacturer's protocol, identified by Giemsa staining, and subsequently maintained in RPMI 1640 medium for further experiments.

### 4.5 Cell Migration Assay

Cell migration was evaluated using a Transwell system. Briefly, 2.0 × 10^5^ endothelial cells were seeded into the upper chamber and allowed to adhere. Then, 5 × 10^5^ PC12 cells along with nanoparticle‐containing medium were introduced into the same chamber, while the lower compartment was filled with complete medium supplemented with or without 100 µm fMLP. After 24 h of incubation, migrated cells were quantified by flow cytometry.

### 4.6 Cellular Uptake Analysis

PC12 cells in the logarithmic growth phase were plated in 6‐well plates at 1 × 10^6^ cells/mL. Following attachment, 1 mL of different nanoparticle formulations was added to each well. After 6 h of incubation, cells were harvested, resuspended in 500 µL PBS, and analyzed via flow cytometry.

### 4.7 Measurement of Mitochondrial Membrane Potential

Treated cells were washed with PBS and incubated with JC‐1 staining solution (diluted 1:1 in culture medium) at 37°C for 20 min. After washing with pre‐cooled JC‐1 buffer, fluorescence was observed and imaged under an inverted fluorescence microscope.

### 4.8 MCAO/R Model

Male C57BL/6 mice (6–8 weeks old, 20 ± 2 g) were subjected to transient focal cerebral ischemia via intraluminal filament occlusion of the right middle cerebral artery. After 90 min of occlusion, the filament was withdrawn to initiate reperfusion for 6 h. The mice were anesthetized with a low concentration of isoflurane, and a constant temperature blanket was used to maintain their body temperature during the surgery. Neurological deficits were assessed using the Zea Longa score; animals with scores of 1–3 were included in the study, whereas those with unsuccessful occlusion or mortality were excluded.

After 30 min of reperfusion, the mice were randomly assigned to the treatment groups and were given PBS, Mdivi‐1, M@Mdivi‐1, or NeuM@Mdivi‐1 (1 mg/kg) via the tail vein for continuous administration for 3 days. The Sham group was given PBS by tail vein injection.

### 4.9 Intracerebral Hemorrhage (ICH) Model in Piglets

Male piglets (8 weeks old, 13–15 kg) were anesthetized with ketamine (20 mg/kg, i.m.) and maintained on isoflurane. Under physiological monitoring, a 3 cm scalp incision was made along the sagittal suture, and a 2 mm circular hole was drilled approximately 15 mm from the sagittal suture, 5 mm anterior to the right coronal suture, and 5 mL of autologous blood collected from the femoral artery was infused slowly into the right basal ganglia to induce ICH.

The sham group underwent only cranial drilling without autologous blood injection and received an equivalent volume of normal saline intraperitoneally; the ICH group received normal saline intraperitoneally at 30 min post‐ICH induction, repeated every 24 h for 2 days; and the NeuM@Mdivi‐1 group received 5 mg/kg NeuM@Mdivi‐1 intraperitoneally at 30 min post‐ICH induction, repeated every 24 h for 2 days.

### 4.10 Neurofunctional Assessment

Neurological function in mice was evaluated using the Longa score and rotarod test. Piglets were assessed with the Purdy score. Mice were pretrained on the rotarod apparatus, and latency to fall was recorded as a measure of motor coordination.

### 4.11 In Vivo Biodistribution of Nanoparticles

Cy5‐labeled formulations were administered intravenously to MCAO/R mice. Fluorescence signals in the brain and major organs were monitored over time using an in vivo imaging system. A 630 nm light source was used as the excitation source, and for in vivo spectral imaging, each image was exposed for 100 ms at 1050 LP, and region‐of‐interest (ROI) analysis was performed to quantify accumulation. At specified time intervals, the mice were euthanized, major organs (liver, brain, kidney, spleen, heart, and lungs) were collected, and fluorescence was analyzed using an in vitro imaging system.

### 4.12 TTC Staining for Infarct Volume

Brains were sectioned coronally and stained with 2% TTC at 37°C for 20 min. Infarct areas (unstained white regions) were measured using ImageJ, and infarct volumes were calculated accordingly.

### 4.13 Evans Blue Extravasation Assay

BBB permeability was assessed by intravenous injection of 2% Evans blue. After 24 h, mice were perfused transcardially, and Evans blue content in brain homogenates was quantified spectrophotometrically.

### 4.14 MRI imaging

Mice subjected to MCAO (n = 3 per treatment group) were anesthetized with isoflurane. T2‐weighted coronal magnetic resonance imaging (MRI) of the mouse brains was subsequently performed using a 3.0 T MR scanner (Siemens Trio, Germany) and a 7.0 T CG NOVILA system (Shanghai Chenguang Medical Technology Co., Ltd., China). MRI scanning parameters were optimized as required, and the acquired images were imported into 3D Slicer software for subsequent measurement and analysis.

### 4.15 Ultrastructural Analysis by TEM

Brain tissue samples were fixed overnight at 4°C with 4% glutaraldehyde, according to the previously described protocol [34]. After removal of the fixative, the tissues were washed three times with phosphate‐buffered saline (PBS), followed by dehydration, embedding, and sectioning. Ultrastructural observation was carried out using a transmission electron microscope (Hitachi, Japan, model HT7700).

### 4.16 Immunofluorescence Staining

Cells were seeded uniformly in confocal dishes at a density of 2.5 × 10^5^ cells/mL. After treatment, they were fixed with 4% paraformaldehyde for 15 min, permeabilized with 0.3% Triton X‐100 for 10 min, and blocked with 10% goat serum at room temperature for 2 h. The cells were then incubated with primary antibodies at 4°C overnight. After washing three times with PBS, the corresponding secondary antibodies were applied. Nuclei were counterstained with DAPI‐containing anti‐fade mounting medium for 10 min at room temperature. For tissue sections, a similar immunofluorescence staining procedure was employed. Images were acquired using a laser scanning confocal microscope.

### 4.17 mtDNA Copy Number Quantification

Total DNA was extracted from cells and brain tissues using a commercial DNA extraction kit (Tiangen, Beijing, China) according to the manufacturer's instructions. mtDNA copy number was quantified by real‐time quantitative polymerase chain reaction (RT‐qPCR), using the nuclear‐encoded β‐actin gene as the endogenous reference and the mitochondrial‐encoded ND1 gene as the target. The primer sequences are listed in Table S1. Amplification was performed on a real‐time PCR system, and the relative mtDNA copy number was calculated using the 2^–ΔΔCt method.

### 4.18 Western Blot Analysis

Total protein was extracted from the samples, and the protein concentration was determined using the BCA assay. Subsequently, 30 µg of protein from each sample was separated by SDS‐PAGE and transferred onto a membrane. The membrane was then blocked with 5% skim milk for 2 h at room temperature with gentle shaking. The following primary antibodies were applied at the indicated dilutions: Drp1 (1:500, Proteintech), Fis1 (1:500, Proteintech), OPA1 (1:1000, Proteintech), Mfn2 (1:1000, Proteintech), GPX4 (1:500, Proteintech), SLC7A11 (1:1000, Proteintech), TBK1 (1:500, Proteintech), p‐TBK1 (1:500, Proteintech), IRF3 (1:5000, Proteintech), and p‐IRF3 (1:1000, Proteintech). The membrane was incubated with primary antibodies overnight at 4°C with shaking. After washing with TBST, a horseradish peroxidase‐conjugated secondary antibody (1:5000, Abcam) was applied and incubated for 2 h at room temperature. Following another TBST wash, protein bands were visualized using ECL chemiluminescence and captured with a gel imaging system. Band intensity was quantified using ImageJ software, and the relative expression of each target protein was normalized to that of the corresponding internal control.

### 4.19 ELISA

Levels of ATP, Fe^2+^, IL‐6, TNF‐α, and IFN‐γ were measured in cell or tissue lysates using commercial ELISA kits according to the manufacturer's protocols.

### 4.20 RNA Sequencing

Brain tissues were collected and sent to Sangon Biotech (Shanghai, China) for RNA extraction and sequencing. Subsequent bioinformatic analysis and data mining were performed using the Sangerbox platform (https://sangerbox.com).

### 4.21 Statistics and Reproducibility

Data are presented as mean ± standard deviation (SD). Normalization and transformation methods are listed in the legends of the respective figures, along with the number of animals per group and the number of in vitro replicates for each experiment. Statistical analyses were performed using GraphPad Prism (version 10.4.2). Comparisons between two groups were made using two‐tailed unpaired Student's *t*‐tests. For comparisons involving three or more groups, differences were assessed by one‐way or two‐way analysis of variance (ANOVA), followed by Tukey's multiple comparison test (when variances were equal) or Dunnett's multiple comparison test (when variances were unequal). Exact *P*‐values are shown directly in the figures, and a *P*‐value < 0.05 was considered statistically significant.

## Author Contributions

Y.L. and Z.Y. synthesized and characterized the nanomaterials. Y.L. and X.G. conducted animal experiments. F.L., S.T., and Z.Y. collected all the cell experiment data. Y.Z., R.J., and H.W. contributed to the western blot experiments and revised the manuscript. X.L. and Q.C. analyzed the data. C.H. and L.X. edited and revised the manuscript. H.B., J. X., Q.M., and L.T. conceived the project, designed the experiments, and wrote the manuscript.

## Conflicts of Interest

The authors declare no conflict of interest.

## Supporting information




**Supporting File**: advs76859‐sup‐0001‐SuppMat.docx.

## Data Availability

The data that support the findings of this study are available from the corresponding author upon reasonable request.
