# Supplementary material for: Rocket‐Inspired Sequentially Targeted Nanotherapeutics for Mitochondrial Regulation and Inflammatory Reprogramming in Ischemic Stroke
Source: Adv Sci (Weinh). 2026 Jul 27:e76859. Online ahead of print. doi: 10.1002/advs.76859 (PMC13404862; doi:10.1002/advs.76859)
Supplement: Supplementary file 1 — Supporting File: advs76859‐sup‐0001‐SuppMat.docx. [file ADVS-9999-e76859-s001.docx]

***Supplementary Information***

**Rocket-Inspired Sequentially Targeted Nanotherapeutics for Mitochondrial Regulation and Inflammatory Reprogramming in Ischemic Stroke**

*He Bai^#^, Zihao Yong^#^, Yang Li^#^, Qingmin Chen^#^, Yong Liu,* *Xiaomeng Guo**, Fukai Li, Shicheng Tang, Zhuo Yang, Yufei Zhang, Ruwei Jie, Xiaohong Lv, Huacheng Wang, Chunming Huang, Liwei Xie, Jianqi Xiao^*^, Qingchun Mu^*^, Longguang Tang^*^*

H. Bai, Y. Li, L. Tang

Department of Pharmacy, Center for Regenerative and Aging Medicine, the Fourth Affiliated Hospital of School of Medicine, and International School of Medicine, International Institutes of Medicine, Zhejiang University, Yiwu 322000, China

Email: [tanglongguang@zju.edu.cn](mailto:tanglongguang@zju.edu.cn) (L.T.)

Z. Yong, Y. Liu, X. Guo, F. Li, S. Tang, Z. Yang

College of Life Sciences, Mudanjiang Medical University, Mudanjiang 157011, Heilongjiang, P. R. China

R. Jie, C. Huang, Q. Mu,

Affiliated Gaozhou People’s Hospital, Guangdong Medical University, Maoming 525200, Guangdong, China

E-mail: [muq@suda.edu.cn](mailto:muq@suda.edu.cn) (Q.M.)

Q. Chen, X. Lv

Shanxi Province Cancer Hospital/Shanxi Hospital Affiliated to Cancer Hospital, Chinese Academy of Medical Sciences/Cancer Hospital Affiliated to Shanxi Medical University, Taiyuan, Shanxi, China

Y. Zhang, H. Wang

Department of Physiology, School of Basic Medical Sciences, Guilin Medical University, Guilin 541199, Guangxi, China

L. Xie

Faculty of Synthetic Biology, Shenzhen University of Advanced Technology, Shenzhen, 518055, China

J. Xiao

The Affiliated Hospital of Guangdong Medical University, Zhan Jiang 524000, Guangdong, China

Email: [xijiqi@gdmu.edu.cn](mailto:xijiqi@gdmu.edu.cn) (J.X.)

^#^ Thes authors contributed equally.

**Supplementary figures**


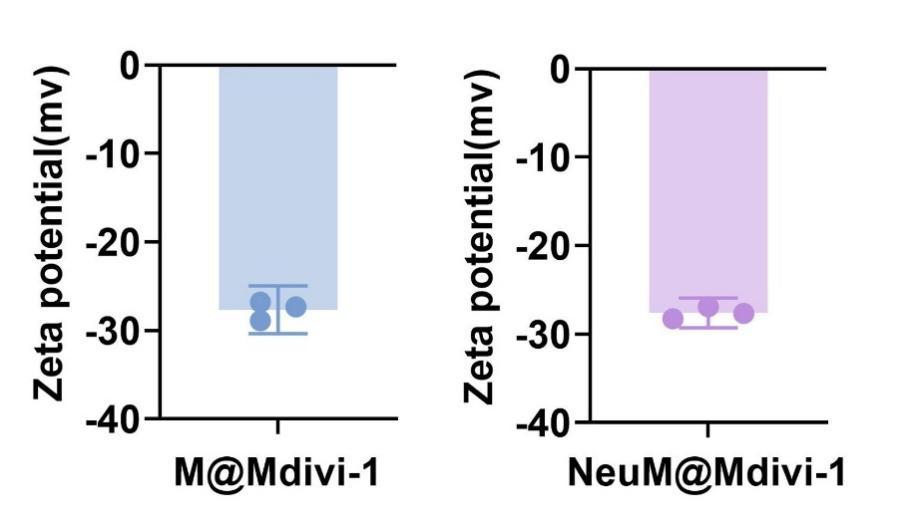


**Figure S1.** The zeta potentials of M@Mdivi-1 and NeuM@Mdivi-1, *n* = 3.


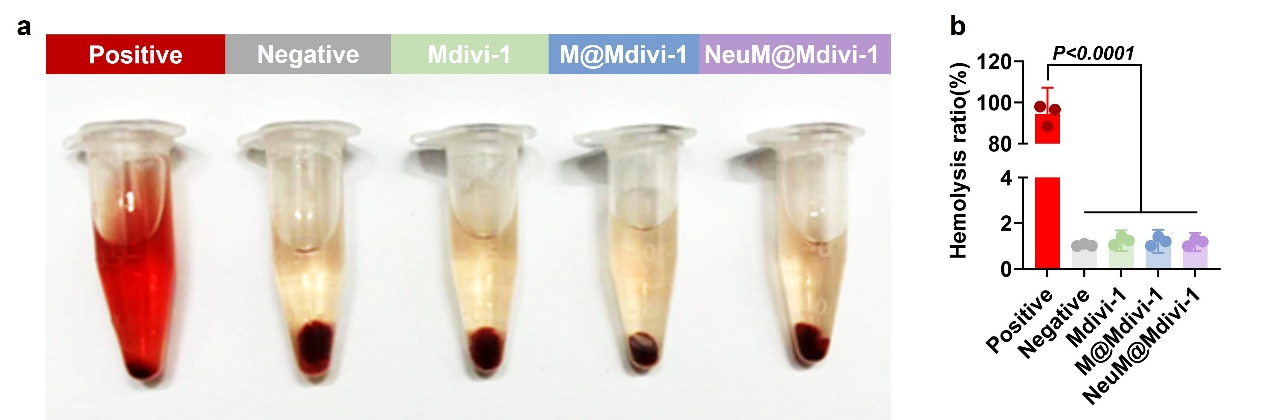


**Figure S2.** Hemolysis analysis of different drugs (0.5 mg/mL). (a) Observation of hemolysis in different groups. (b) Hemolysis rate statistics. Data are presented as the mean ± SD. Statistical significance was analyzed by one-way ANOVA using Tukey’s posttest and the P value marks the significance difference, *n* = 3.


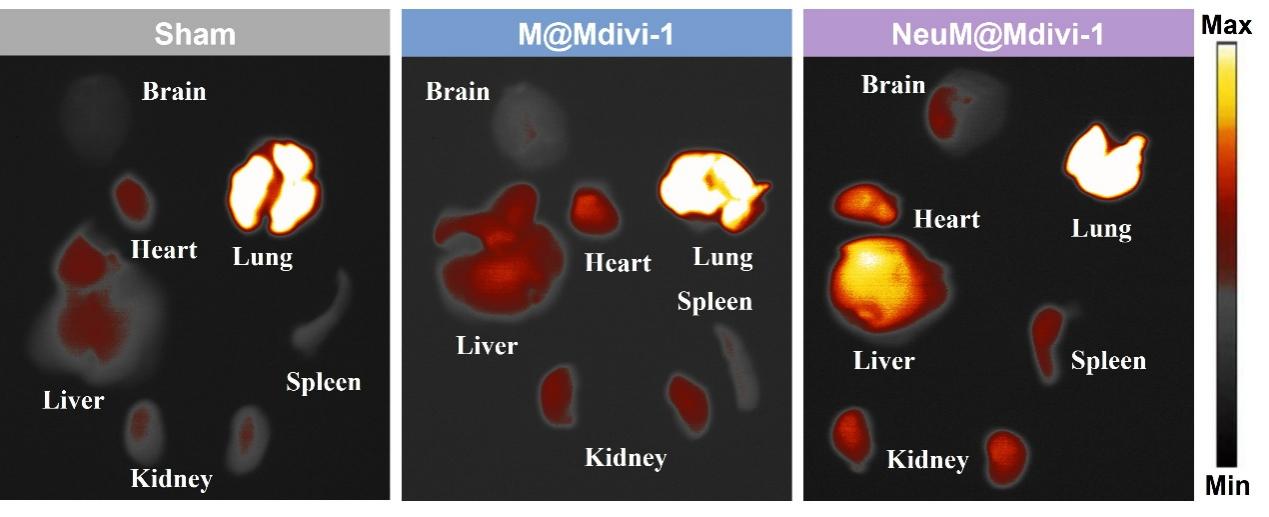


**Figure S3.** NIR-II imaging of isolated tissues after 24 h administration.


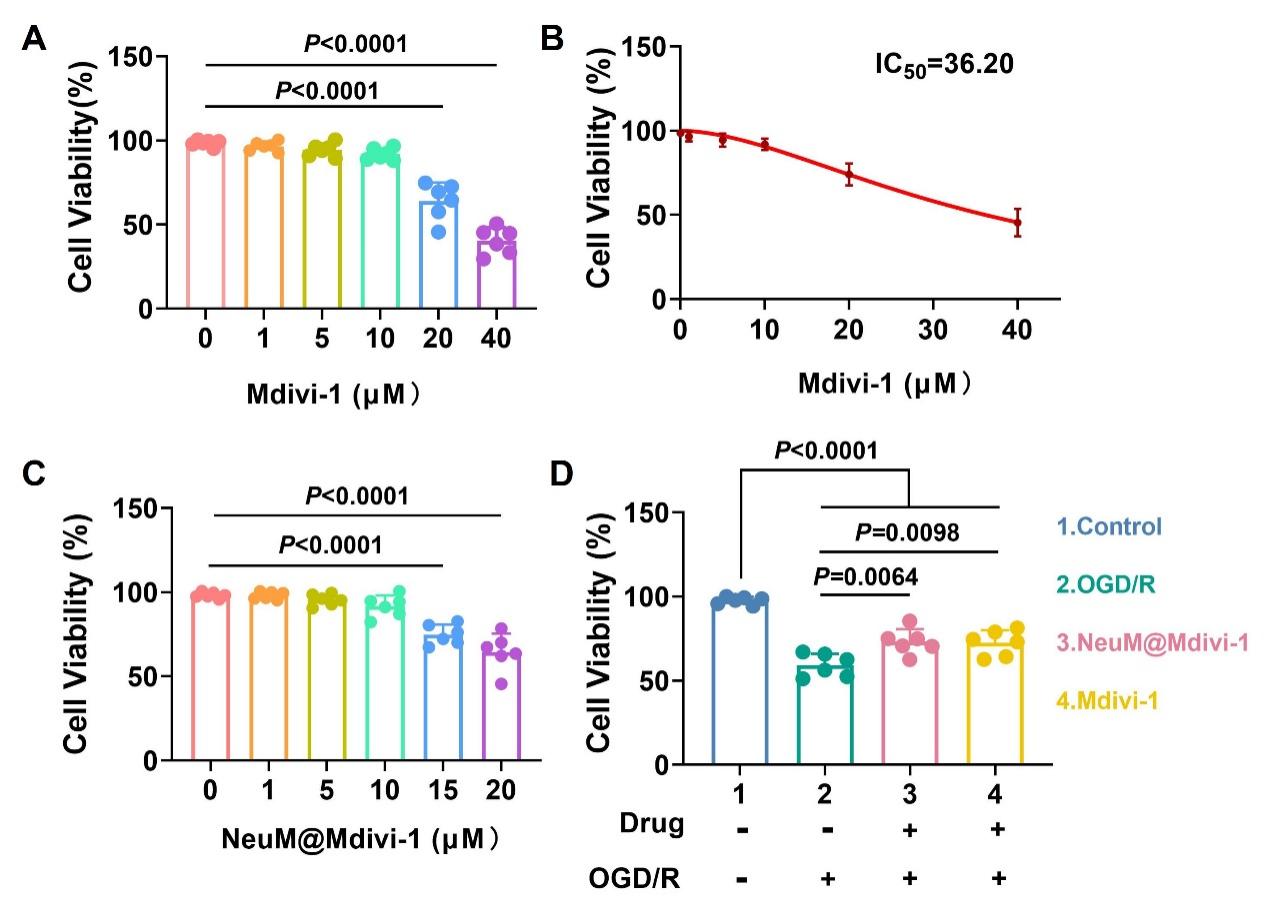


**Figure S4.** The cell survival rate measured by CCK8 after different treatments. (a) Cell survival rate after different concentrations of Mdivi-1 treatment. (b) The median inhibitory concentration of Mdivi-1. (c) Cell survival rate after different concentrations of NeuM@Mdivi-1 treatment. (d) Cell survival rate after OGD/R and NeuM@Mdivi-1 treatment. Data are shown as the mean ± SD. Representative data from one of the experiments are shown. Statistical significance was analyzed by one-way ANOVA using Tukey’s posttest and the *P* value marks the significance difference, *n* = 6.


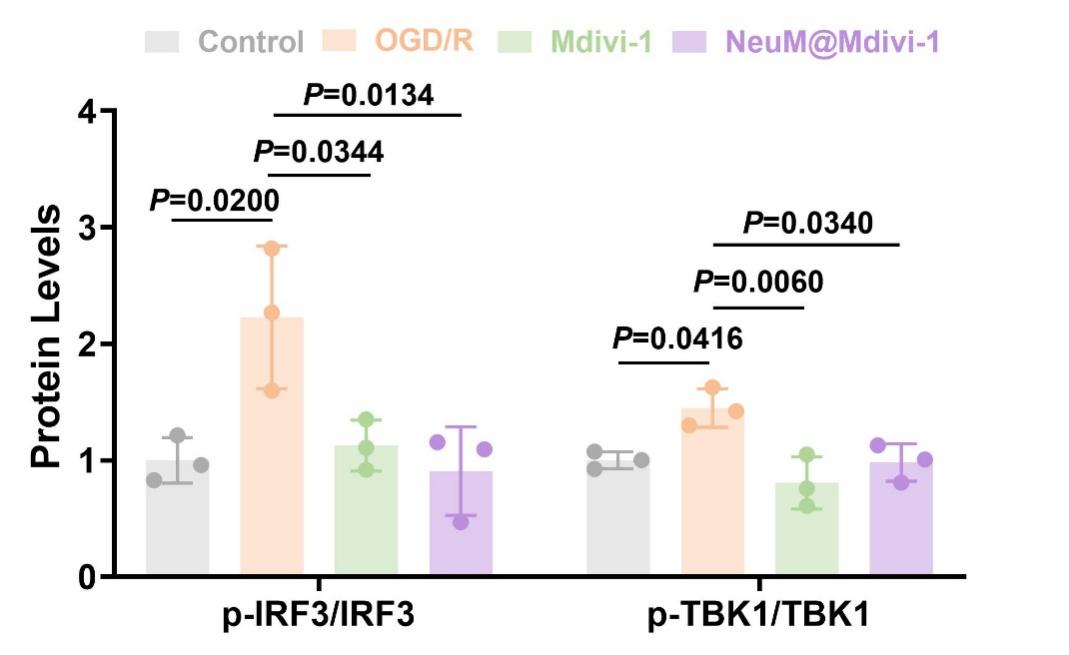


**Figure S5.** Related protein expression levels of STING pathway. The experiments were repeated at least three times and representative data from one of the experiments are shown. Data are presented as the mean ± SD. Statistical significance was analyzed by one-way ANOVA using Tukey’s posttest and the *P* value marks the significance difference, *n* = 3.


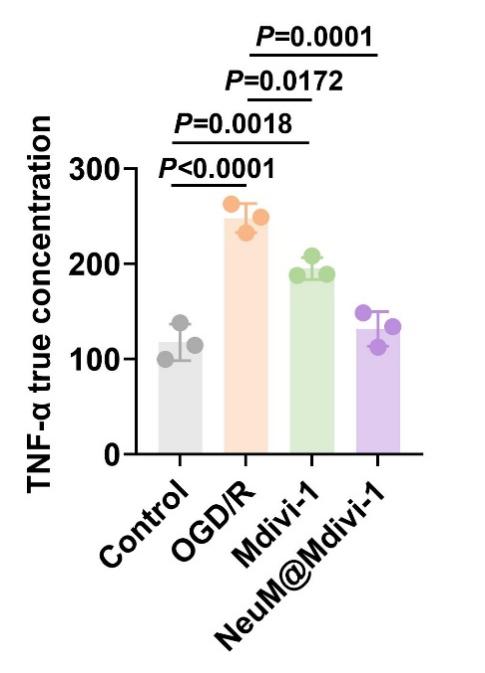


**Figure S6.** The content of TNF-α in different groups. Data are presented as the mean ± SD. Statistical significance was analyzed by one-way ANOVA using Tukey’s posttest and the *P* value marks the significance difference, *n* = 3.


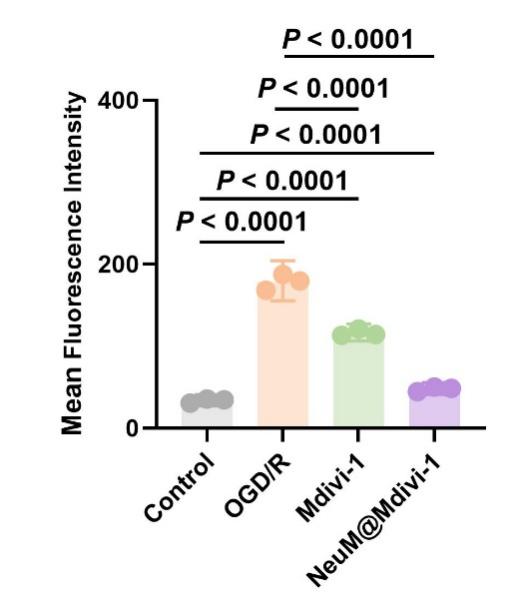


**Figure S7.** The quantitative analysis of Fe^2+^. Data are presented as the mean ± SD. Statistical significance was analyzed by one-way ANOVA using Tukey’s posttest and the *P* value marks the significance difference, *n* = 3.


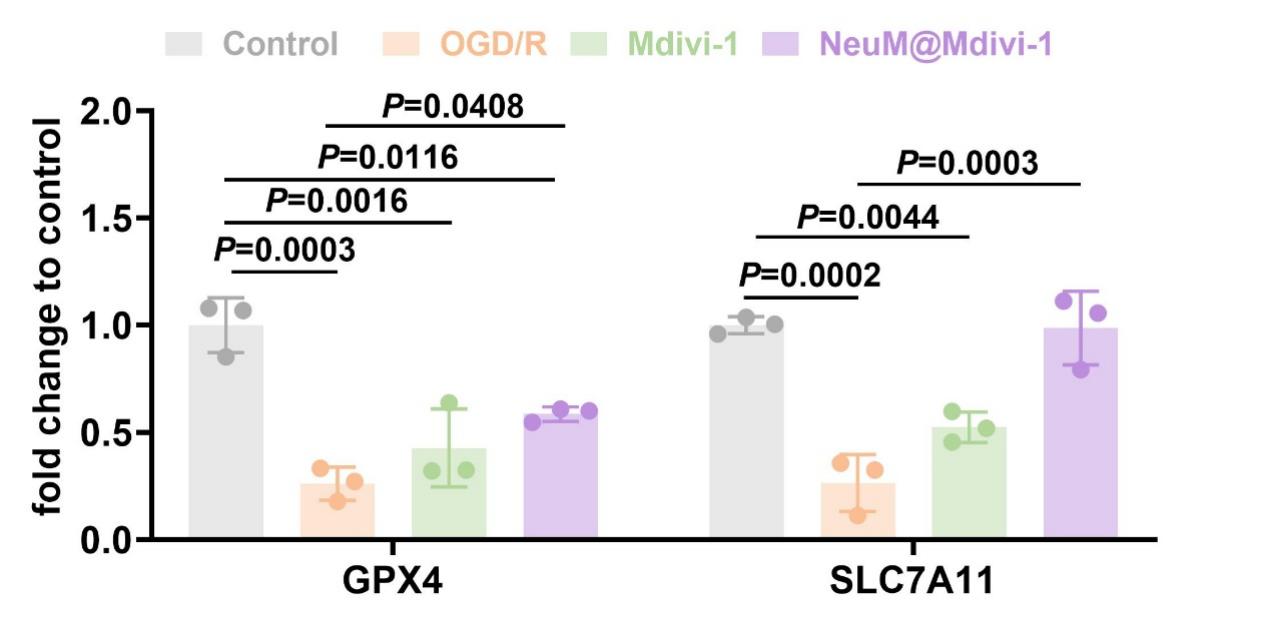


**Figure S8.** Related protein expression levels of ferroptosis pathway. The experiments were repeated at least three times and representative data from one of the experiments are shown. Data are presented as the mean ± SD. Statistical significance was analyzed by one-way ANOVA using Tukey’s posttest and the *P* value marks the significance difference, *n* = 3.


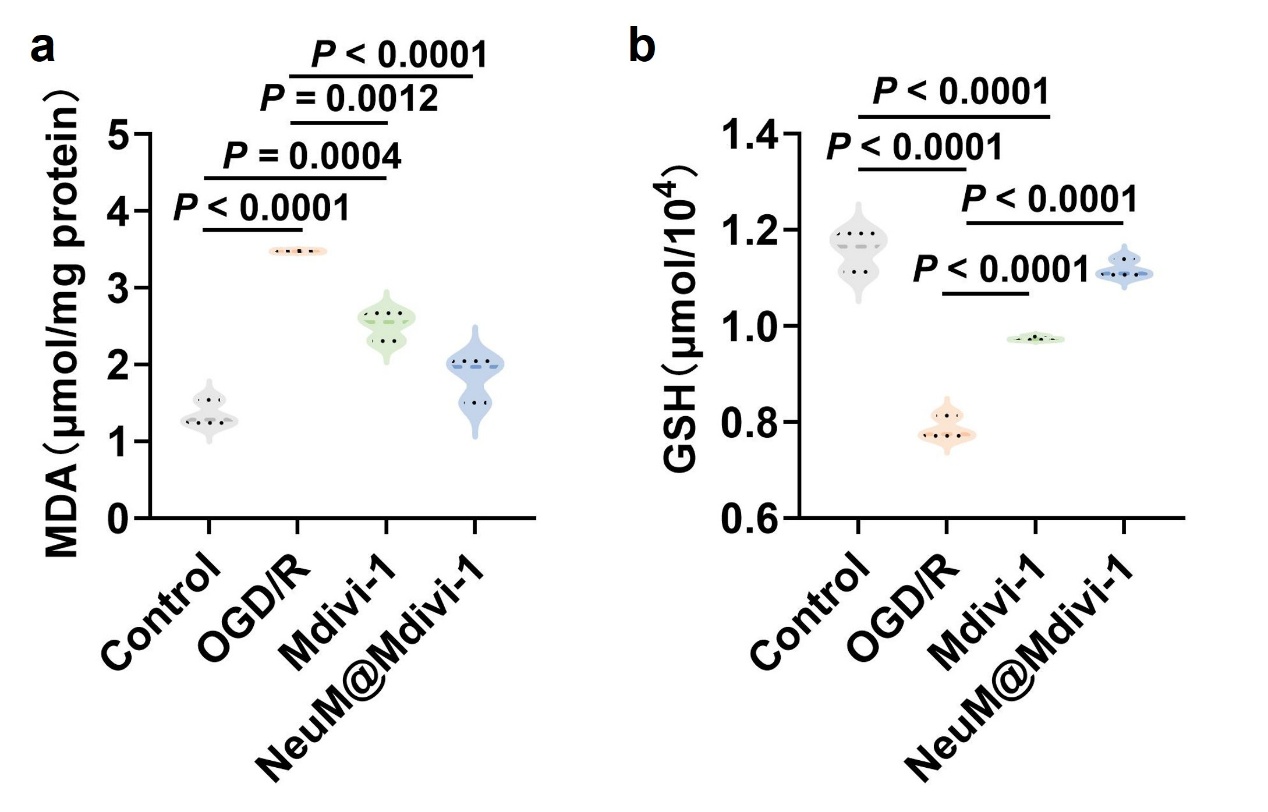


**Figure S9.** (a)The content of GSH in different groups. (b) The content of MDA in different groups. Data are presented as the mean ± SD. Statistical significance was analyzed by one-way ANOVA using Tukey’s posttest and the *P* value marks the significance difference, *n* = 3.


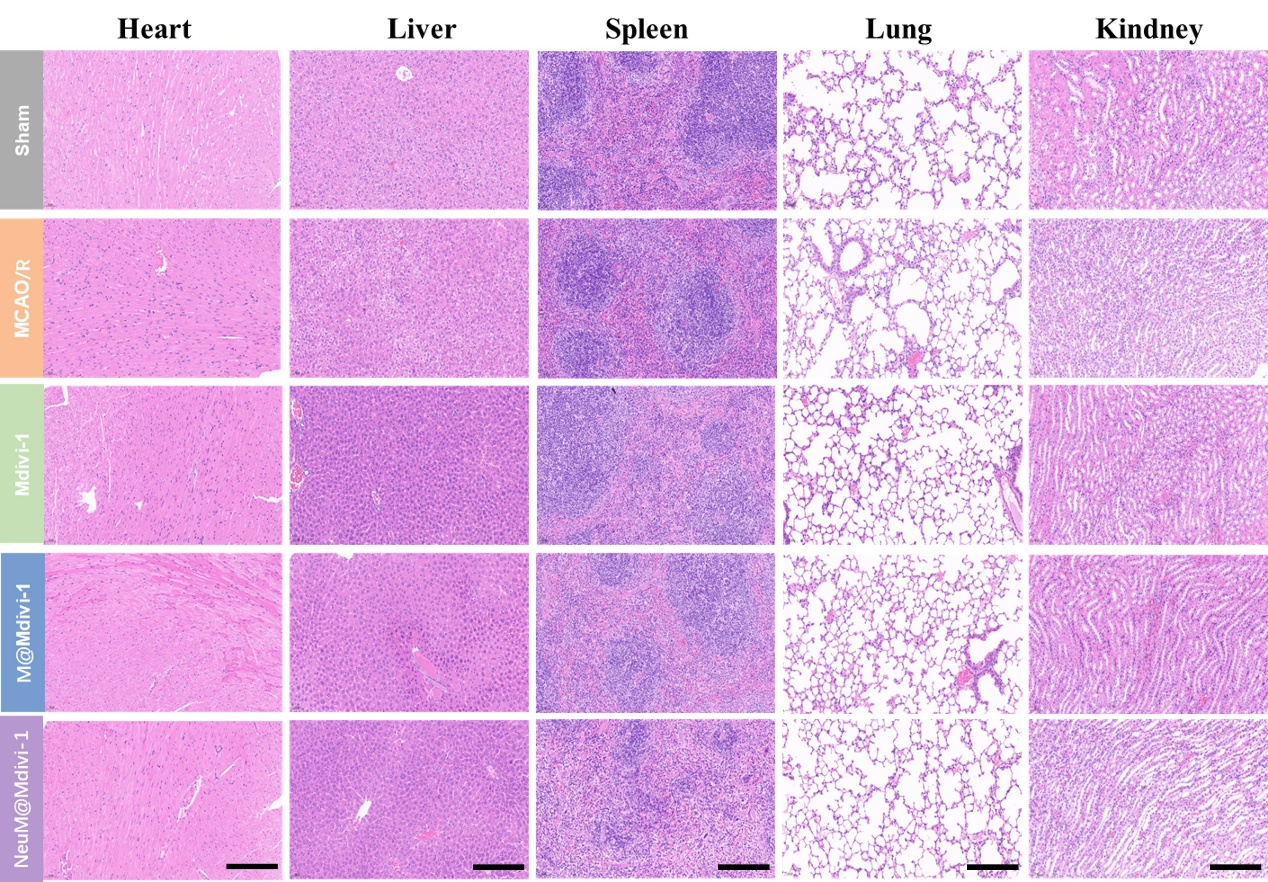


**Figure S10.** HE staining pathological sections of the heart, liver, spleen, lung and kidney after treatment with different liposomes. Scale bar = 200 μm.


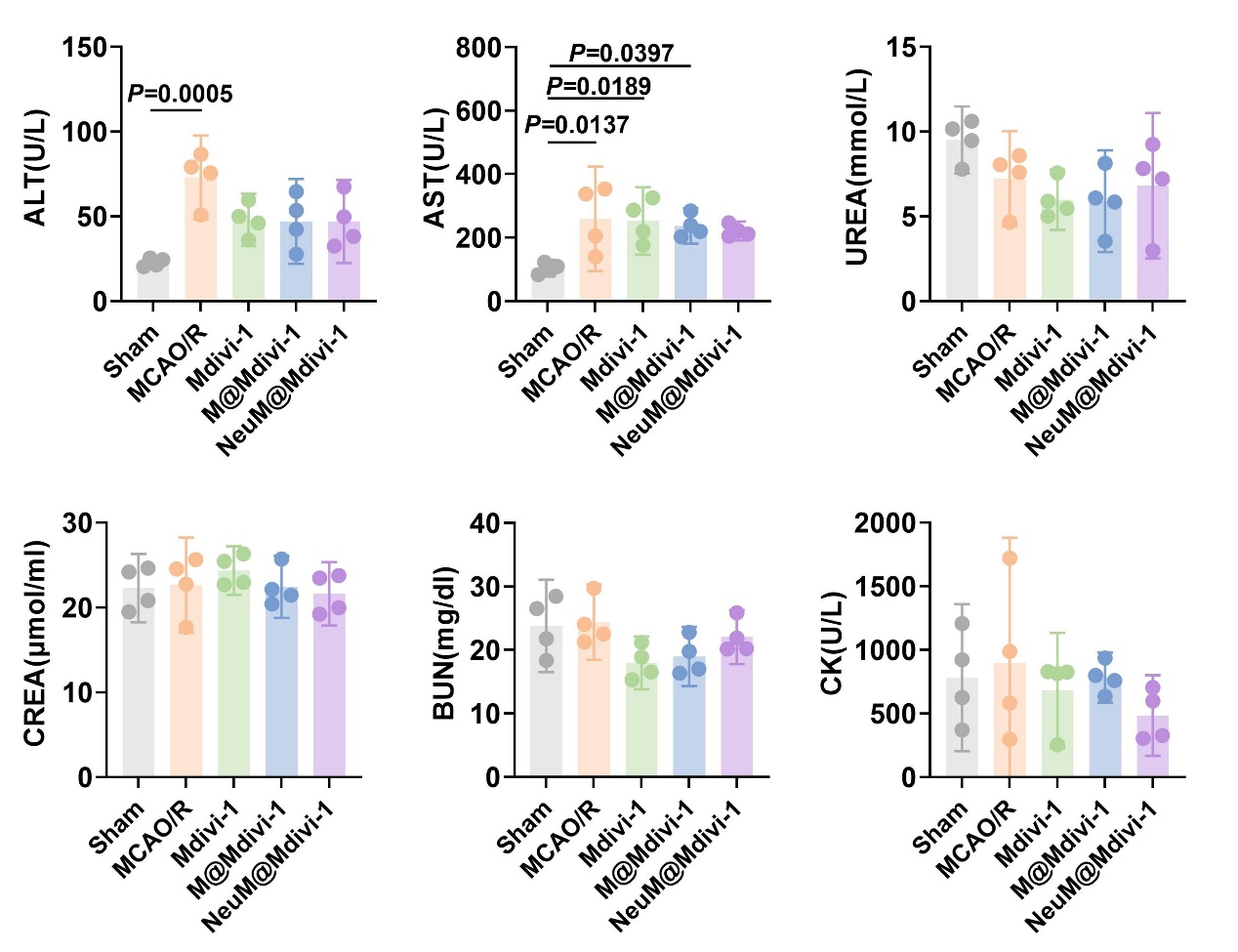


**Figure S11.** Serum biochemical parameters of mice in different treatment groups (*n* = 3 mice in each group). Data are shown as the mean ± SD. Representative data from one of the experiments are shown. Statistical significance was analyzed by one-way ANOVA using Tukey’s posttest and the *P* value marks the significance difference, *n* = 3.


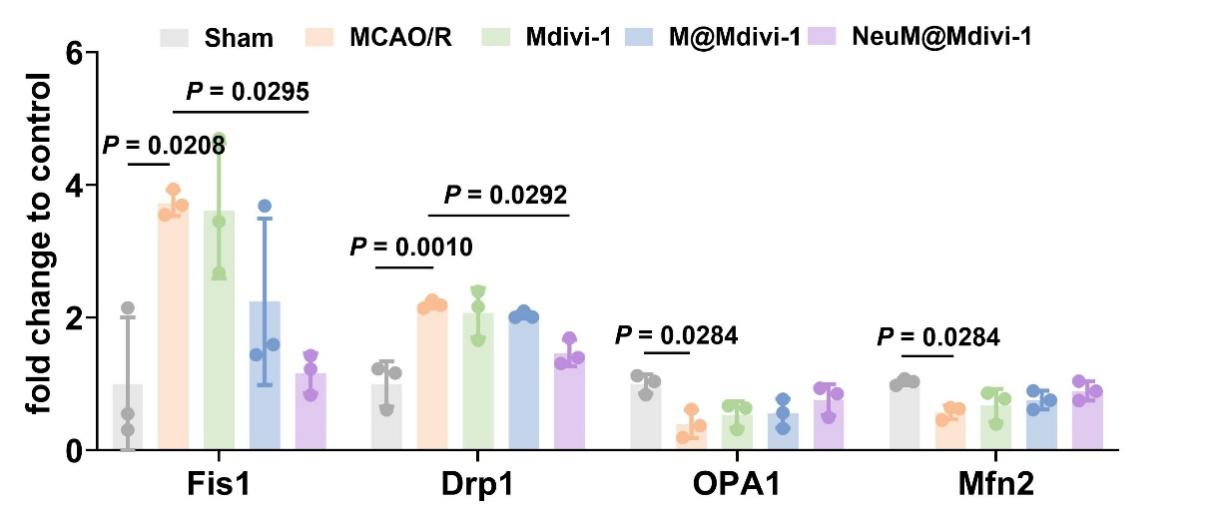


**Figure S12.** Related protein expression levels of mitochondrial fission and fusion in mice. The experiments were repeated at least three times and representative data from one of the experiments are shown. Data are presented as the mean ± SD. Statistical significance was analyzed by one-way ANOVA using Tukey’s posttest and the *P* value marks the significance difference, *n* = 3.


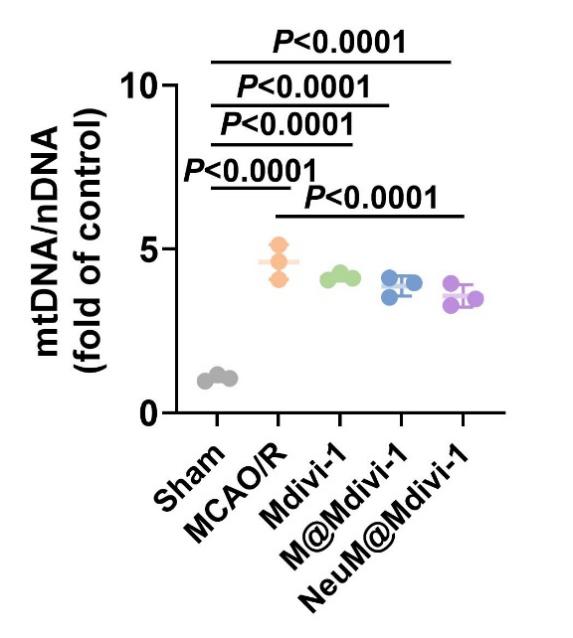


**Figure S13.** mtDNA copy number (*n* = 3 mice in each group). Data are presented as the mean ± SD. Statistical significance was analyzed by one-way ANOVA using Tukey’s posttest and the *P* value marks the significance difference.


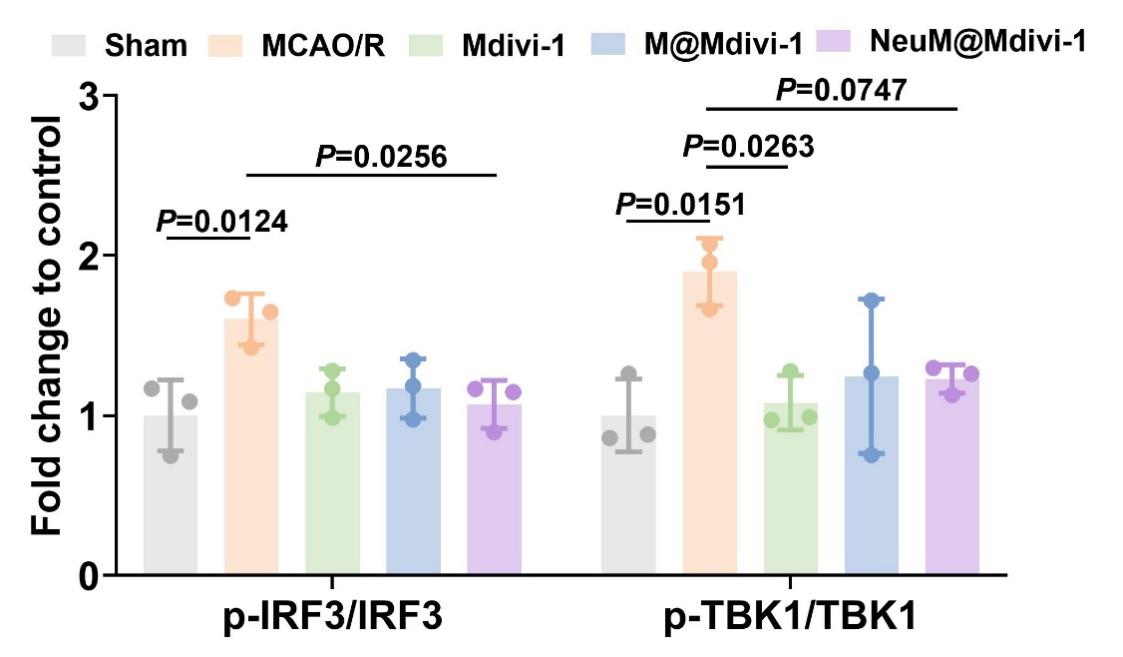


**Figure S14.** Related protein expression levels of STING pathway in mice. The experiments were repeated at least three times and representative data from one of the experiments are shown. Data are presented as the mean ± SD. Statistical significance was analyzed by one-way ANOVA using Tukey’s posttest and the *P* value marks the significance difference, *n* = 3.


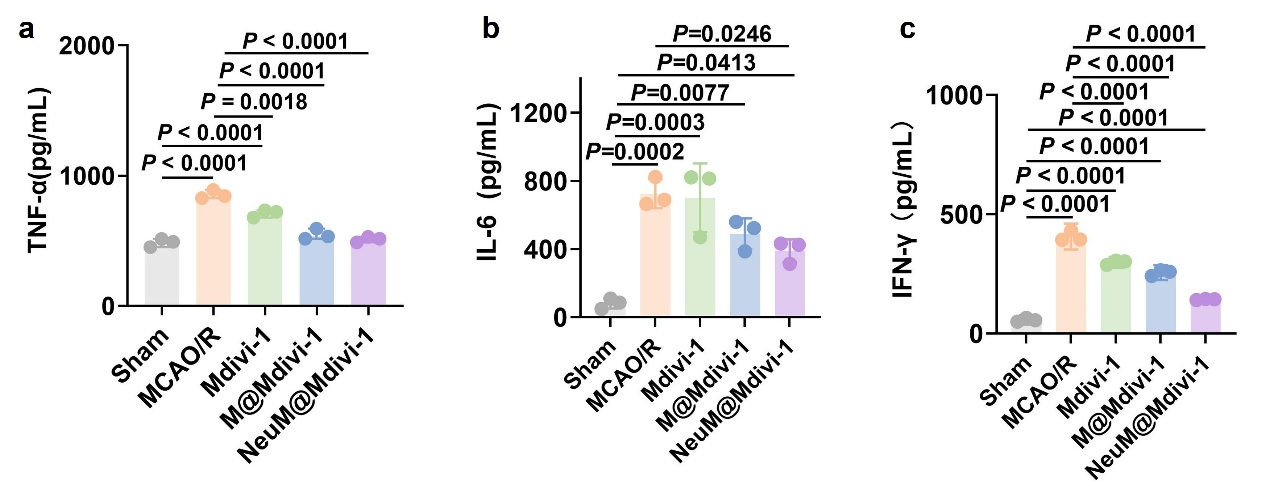


**Figure S15.** Inflammatory-related indicators in mouse serum (n = 3 mice in each group). (a) The content of TNF-α. (b) The content of IL-6. (c) The content of IFN-γ. Data are presented as the mean ± SD. Statistical significance was analyzed by one-way ANOVA using Tukey’s posttest and the *P* value marks the significance difference, *n* = 3.


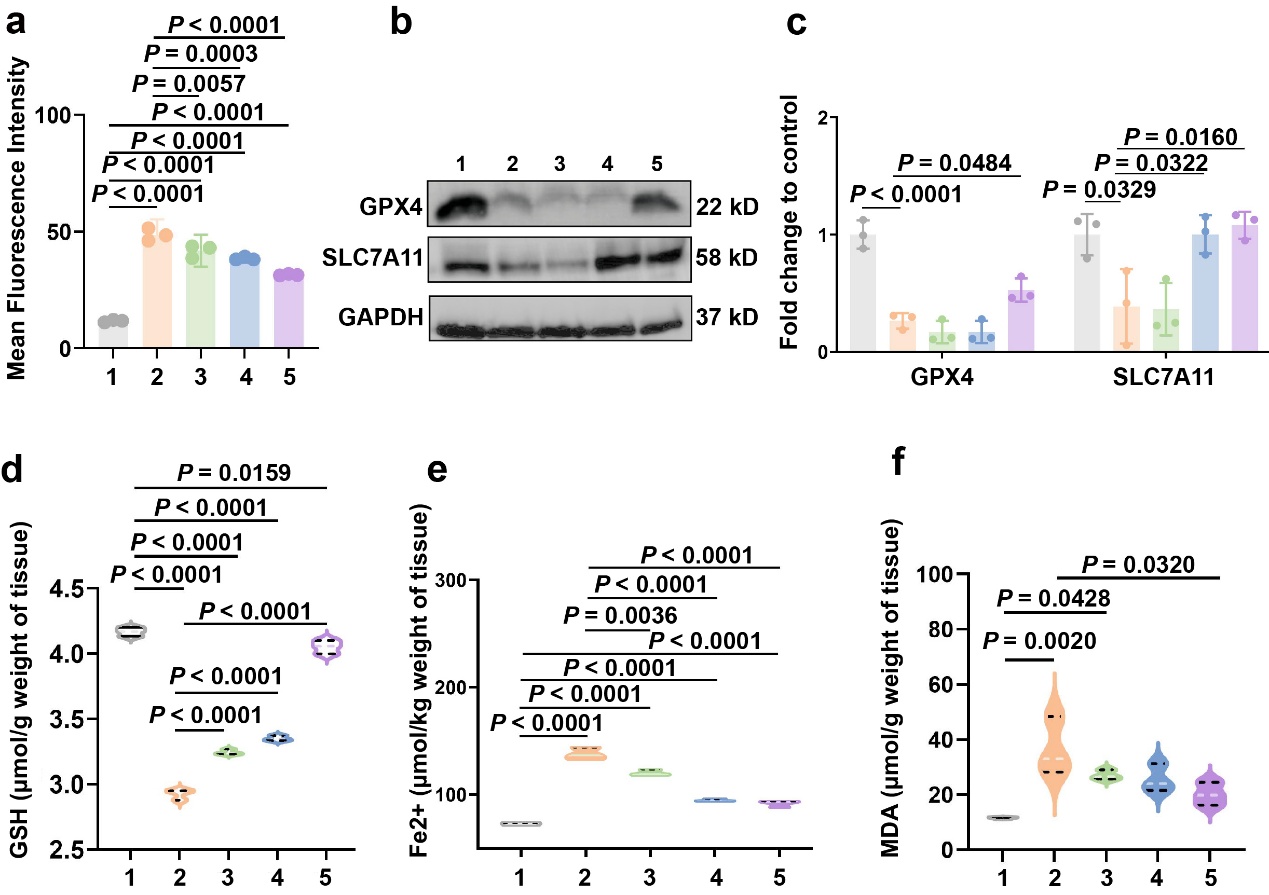


**Figure S16.** Data related to ferroptosis in mice (n = 3 mice in each group). (a) The quantitative analysis of 4-HNE. (B) Related protein expression levels of ferroptosis. (c) Quantitative analysis of protein expression. (d) The content of GSH. (e) The content of Fe^2+^. (f) The content of MDA. Data are presented as the mean ± SD. Statistical significance was analyzed by one-way ANOVA using Tukey’s posttest and the *P* value marks the significance difference, *n* = 3.

**Table S1 Primer sequences**

| Gene name | Sequence (5 '→3') | Entry number | Species |
| --- | --- | --- | --- |
| ND1 | Forward:  ACTTCCCAGCATGTGCTTCA | AB055739.1 | Mouse |
|  | Reverse:  CGGTGTGCCATAAAATGCGT |  |  |
| β-actin | Forward: AGGGAAATCGTGGTGGTGACAT | AY618569.1 | Mouse |
|  | Reverse: GGAAAAGCCCCAGGAGGGCAT |  |  |
| ND1 | Forward: ACAACCAATAGCAGACGCAC | MG963139.1 | Rat |
|  | Reverse: GAAATGGTTTGGGCTACGGC |  |  |
| β-actin | Forward: CACCGCAAATGCTTCTAGGC | V01217.1 | Rat |
|  | Reverse: CTTTGGGGGATGTTTGCTCCA |  |  |
